# Immune exhaustion in chronic Chagas disease: Pro-inflammatory and immunomodulatory action of IL-27 *in vitro*

**DOI:** 10.1371/journal.pntd.0009473

**Published:** 2021-06-01

**Authors:** María Ailén Natale, Todd Minning, María Cecilia Albareda, Melisa Daiana Castro Eiro, María Gabriela Álvarez, Bruno Lococo, Gonzalo Cesar, Graciela Bertocchi, María Josefina Elias, María Belén Caputo, Rick Lee Tarleton, Susana Adriana Laucella

**Affiliations:** 1 Instituto Nacional de Parasitología Dr. Mario Fatala Chaben, Buenos Aires, Argentina; 2 Consejo Nacional de Investigaciones Científicas y Técnicas (CONICET), Buenos Aires, Argentina; 3 Center for Tropical and Emerging Global Diseases, University of Georgia, Athens, Georgia, United States of America; 4 Hospital Interzonal General de Agudos “Eva Perón”, San Martín, Argentina; University of Texas at El Paso, UNITED STATES

## Abstract

In chronic Chagas disease, *Trypanosoma cruzi*-specific T-cell function decreases over time, and alterations in the homeostatic IL-7/IL-7R axis are evident, consistent with a process of immune exhaustion. IL-27 is an important immunoregulatory cytokine that shares T-cell signaling with IL-7 and other cytokines of the IL-12 family and might be involved in the transcriptional regulation of T-cell function. Here, we evaluated the expression and function of IL-27R in antigen-experienced T cells from subjects with chronic Chagas disease and assessed whether *in vitro* treatment with IL-27 and IL-7 might improve *T*. *cruzi*-specific polyfunctional T-cell responses. *In vitro* exposure of PBMCs to *T*. *cruzi* induced a downregulation of IL-27R in CD4^+^ T cells and an upregulation in CD8^+^ T cells in subjects without heart disease, while IL-27R expression remained unaltered in subjects with more severe clinical stages. The modulation of IL-27R was associated with functional signaling through STAT3 and STAT5 and induction of the downstream genes *TBX21*, *EOMES* and *CXCL9* in response to IL-27. *In vitro* treatment of PBMCs with IL-27 and IL-7 improved monofunctional and polyfunctional Th1 responses, accompanied by the induction of IL-10 and Bcl-2 expression in subjects without heart disease but did not improve those in subjects with cardiomyopathy. Our findings support the process of desensitization of the IL-27/IL-27R pathway along with disease severity and that the pro-inflammatory and immunomodulatory mechanisms of IL-27 might be interconnected.

## Author summary

Chagas disease, caused by the intracellular protozoan parasite *Trypanosoma cruzi*, affects approximately 10 million people worldwide. In 20%–30% of infected individuals, the disease results in heart disease or mega esophagus/megacolon, making Chagas disease the most common cause of infectious myocarditis in the world. Previous studies have shown that the immune response specific for this parasite becomes impaired over time. One mechanism that could explain the loss of immune cells is the defective signaling of cytokines involved in the maintenance of T cells. In this work, we evaluated the signaling pathways of IL-27 and IL-7, which promote T-cell differentiation, but IL-27 might also exert immunomodulatory actions. We found alterations in the IL-27/IL-7 pathways in chronic Chagas disease that affect not only the signal-transduction level but also the transcriptional regulation level. *In vitro* treatment of PBMCs of patients in early clinical stages of chronic Chagas disease could restore T-cell function, but this was not possible in cell cultures from patients with severe heart disease in whom the immune system is deeply exhausted. These findings contribute to a better understanding of the immune mechanisms underlying the anti-*T*. *cruzi* response in human subjects which might be useful for vaccine and new therapeutic interventions.

## Introduction

*Trypanosoma cruzi*, an intracellular protozoan parasite, is the causative agent of Chagas disease, and it affects approximately six million people from the southern U.S. to South America and Western Europe [1,2]. The persistence of parasites is regarded as the primary cause of cumulative tissue damage in chronic Chagas disease [3]. Following an acute infection, T cells typically coexpress multiple cytokines including interleukin-2 (IL-2), tumor necrosis factor-α (TNF-α), interferon-γ (IFN-γ), and cytotoxic effector molecules—a phenotype that is often described as polyfunctional [4]. However, when infections become chronic, a consecutive loss of T-cell functions along with the expression of inhibitory receptors occurs through a process known as T-cell exhaustion, resulting in a decrease in the levels of pathogen-specific polyfunctional T cells [4]. T helper 1 (Th1) and T cytotoxic (Tc) cells are critical for *T*. *cruzi* immunity [5] and are impaired over time [6,7]. *T*. *cruzi*-infected children, who are presumed to have shorter-term infections compared with adults, showed a higher proportion of polyfunctional CD4^+^ T cells responsive to *T*. *cruzi* antigens in their circulation [7] relative to chronic infected adults [7–9]. The impairment of *T*. *cruzi*-specific T-cell function is more pronounced in patients with advanced cardiomyopathy [6,8,10]. Defects in immunoregulatory pathways have also been implicated in the progression of cardiac disease [11–13].

Defects in T-cell signaling appear as one of the mechanisms by which T-cell responses can be abrogated during persistent infections [4]. We have shown that the levels of IFN-γ-producing cells in response to *T*. *cruzi* antigens are positively associated with the expression and function of the receptor of homeostatic IL-7 in T cells and can be augmented after *in vitro* stimulation with IL-7 or IL-27 [14]. IL-27 can mediate the differentiation and proliferation of Th1 cells [15], activation of cytotoxic CD8^+^ T lymphocytes [16] and NK cells [17], and suppression of Th2 [18]. However, IL-27R signaling might also exert anti-inflammatory effects during T-cell responses by inhibiting the development of Th17 cells [19,20], inducing IL-10 production by CD4^+^ T cells [21] and IL-10-producing regulatory T cells [22], as well as the expression of Tim-3 and PD-L1 in CD4^+^ and CD8^+^ T cells [23]. IL-27, which belongs to the IL-12 cytokine family, exerts its function through the heterodimeric IL-27 receptor (IL-27R) comprising the specific IL-27Rα (WSX-1) and gp130 (CD130) proteins [24] and, similar to IL-7, through the JAK1/2-dependent phosphorylation of STAT1, STAT3 and STAT5 [18,25,26]. IL-27 is mostly secreted by activated antigen-presenting cells, and whereas the gp130 chain is widely expressed in all tissues, IL-27Rα is restricted mainly to lymphocytes and monocytes [27]. After activation of T cells, the expression of IL-27R increases in CD8^+^ T cells [16]. However, although it is highly expressed in naïve CD4^+^ T cells, WSX-1 expression declines upon Th-cell differentiation [28]. Accordingly, TCR ligation prompts the expression of WSX-1 by CD4^+^ T cells and induces the production of IL-2, a cytokine that inhibits the expression of this receptor [29]. In experimental acute *T*. *cruzi* infection of mice, the deficiency of IL-27 or WSX-1 inhibited the control of parasite burden despite the elevated production of inflammatory cytokines [30–32]. Whereas previous findings of our group have shown decreased IL-27 serum levels in patients with no signs of cardiac dysfunction [14], other authors have reported increased levels of IL27p28 in these patients [32]. However, it is unknown whether the signaling pathway of IL-27/IL-27R is functional in the chronic phase of Chagas disease in humans. In this work, we sought to evaluate the expression and function of IL-27R in peripheral T cells in patients with chronic Chagas disease with different degrees of cardiac dysfunction and to assess whether *T*. *cruzi*-specific polyfunctional T-cell responses are modified by *in vitro* treatment with IL-27 and IL-7. The gene expression of transcription factors of the IL-27 and IL-7 axis was also evaluated in response to these cytokines.

## Materials and methods

### Ethics statement

This study was approved by the Institutional Review Board of the Hospital Interzonal General de Agudos Eva Perón, Buenos Aires, Argentina. All patients signed informed consent forms prior to inclusion in the study.

### Selection of study population

Subjects were recruited at the Chagas Disease Unit, Cardiology Department, Hospital Interzonal General de Agudos Eva Perón, Buenos Aires, Argentina. Patients who tested positive for two out of three serological tests for *T*. *cruzi* infection (i.e., indirect immunofluorescence assays, hemagglutination and ELISA tests) were considered to be infected [33]. *T*. *cruzi*-infected subjects were originally infected while living in areas endemic for *T*. *cruzi* infection but had lived in Buenos Aires, which is a nonendemic area, for a minimum of 15 years. Subjects were clinically evaluated and grouped according to a modified version of the Kuschnir grading system [34]: group 0 (G0), seropositive individuals exhibiting a normal electrocardiogram (ECG) and normal echocardiograph; group 1 (G1), seropositive individuals with a normal echocardiograph but abnormalities in the ECG; group 2 (G2), seropositive individuals with ECG abnormalities and heart enlargement; and group 3 (G3), seropositive individuals with ECG abnormalities, heart enlargement and clinical or radiological evidence of heart failure. The study population characteristics are summarized in Table 1. Age-matched uninfected subjects from Buenos Aires who have always resided in nonendemic areas and with negative serological findings for *T*. *cruzi* infection served as the uninfected control group (UI).

**Table 1:** Characteristics of study population.

Patient group	n	Age range (median), years	Sex	
			Male	Female
G0	49	26–57 (41)	21	28
G1	14	30–58 (50)	5	9
G2	5	46–70 (51)	3	2
G3	8	35–67 (54)	5	3
Uninfected	23	28–61 (40)	9	14

### Collection of peripheral blood mononuclear cells (PBMCs)

Approximately 50 mL of blood was drawn by venipuncture into heparinized tubes (Vacutainer, BD Biosciences). PBMCs were isolated by density gradient centrifugation with Ficoll-Hypaque medium (GE Healthcare) and resuspended in a volume of RPMI 1640 medium (ThermoFisher Scientific) supplemented with 10% heat-inactivated FBS (NATOCOR) (complete RPMI). The cells were then cryopreserved with an equal volume of 20% DMSO and 80% FBS and stored in liquid nitrogen until use. Due to sample availability the assays were not run for all samples.

### *T*. *cruzi* antigens

Amastigote-enriched protein lysate from the Brazil strain of *T*. *cruzi* was produced by four freeze/thaw cycles followed by sonication as previously reported [10].

### Monoclonal antibodies, data acquisition and gating strategies

The monoclonal anti-human antibodies and dyes used for flow cytometry experiments were the following: Fluorescein (FITC)-conjugated anti-CD45RA (BD, 555488), FITC-conjugated anti-Bcl-2 (BD, 340575), FITC-conjugated anti-CD154 (BD, 555699), Alexa Fluor 488 (AF488)-conjugated anti-pSTAT1 (BD, 560191), Phycoerythrin (PE)-conjugated anti-pSTAT5 (BD, 612567), PE-conjugated anti-CD130 (BD, 555757), PE-Cyanine5 (PE-Cy5) and Allophycocyanin (APC)-Cyanine 7(APC-Cy7)-conjugated anti-CD8 (BD, 555636 and Biolegend, 301016), PE-Cy7-conjugated anti-PD-1 (BD, 561272), PE-Cy7-conjugated anti-MIP-1β (BD, 560687), PE-CF594-conjugated anti-CD57 (BD, 560845), peridinin-chlorophyll proteins (PerCP)- and Pacific Blue (PB)-conjugated anti-CD4 (BD, 347324 and 558116), Alexa Fluor 647 (AF647)-conjugated anti-pSTAT3 (BD, 557815), APC-Cy7-conjugated anti-CD3 (BD, 557832), APC-conjugated anti-WSX1 (R&D Systems, FAB14791A), Brilliant Violet 421 (BV421)-conjugated anti-IL-2 (BD, 562914), BV421-conjugated anti-IL-10 (BD, 564053) and Fixable Viability Stain 510 (FV510) (BD, 564406). Data were acquired on HyperCyAn (Beckman Coulter) and FACS Aria II (BD) flow cytometers and analyzed with FlowJo software (Tree Star). Lymphocytes were gated based on forward scatter and side scatter parameters, followed by forward scatter area *vs*. forward scatter height parameters and side scatter area *vs*. side scatter height for doublet discrimination. The subsequent analyses were performed on viable cells (FV510^—^) and CD3^+^ T cells. According to CD45RA expression in CD4^+^ or CD8^+^ T cells, antigen-experienced (CD45RA^—^) T cells were gated and IL-27R expression was assessed in these populations according to fluorescence minus one control (FMO) (S1 Fig).

### Cell-surface staining for phenotypic analyses

A total of 1.5 × 10^6^ PBMCs were stained with monoclonal antibodies and FV510 in staining buffer (PBS with 4% fetal bovine serum- FBS) for 30 min on ice. Then, the cells were washed and resuspended in PBS containing 2% of paraformaldehyde (PFA), incubated for 15 min and washed again.

### PBMCs and *T*. *cruzi* trypomastigote co-cultures

Two × 10^6^ PBMCs were cocultured with Vero-cell-culture-derived trypomastigotes (Brazil strain) at a ratio of 1:5 cells/parasites in a 24-well plate in complete RPMI at 37°C in 5% CO_2_ and 99% humidity for 48 h. A range ratio of 1:1 to 1:5 cells/parasites was used to establish the appropriate conditions for this assay [35]. When noted, human IL-2 was blocked with neutralizing antibodies (BD, 554562) tested at 5 and 10 μg/mL. The latter concentration was found to be most effective, as recommended by the manufacturers. Then, the cells were washed with staining buffer, and cell-surface staining was performed as described above.

### STATs phosphorylation assay

For setting up STAT phosphorylation assays, the IL-27 concentration was tested at 100 ng/mL and 200 ng/mL for 10, 15, and 20 min; the concentration of 200 ng/mL for 10 min was found to be optimum [16,36]. A total of 2 × 10^6^ PBMCs were cultured overnight in serum-free medium (AIM-V, Invitrogen) followed by a 10-min incubation with 200 ng/mL recombinant human IL-27 (IL-27, R&D Systems) at 37°C and 5% CO_2_. Afterwards, the cells were washed with staining buffer, labeled with anti-cell-surface monoclonal antibodies for 20 min on ice and immediately fixed by adding an equal volume of prewarmed 4% PFA for 10 min at 37°C. After centrifugation and PFA removal by aspiration, the cells were permeabilized by adding 1 mL of 90% ice-cold methanol for 30 min on ice and then washed twice with staining buffer. Last, the cells were incubated at room temperature for 30 min with anti-pSTAT1, pSTAT3 and pSTAT5 monoclonal antibodies. Data were acquired on a Hyper Cyan Beckman Counter flow cytometer (Beckman Coulter) and further analyzed with FlowJo (Tree Star) software. To assess the proportion of T cells with three, two, or one phosphorylated STAT, the Boolean gating function in FlowJo software was used. IL-27-induced phosphorylation was considered positive when the percentage ratios of CD4^+^ and CD8^+^ T cells with phosphorylated STAT following stimulation with IL-27 to those in unstimulated cultures was > 50%. Subsequently, the total positive STAT phosphorylation response was calculated for each subject, and the proportion of each subset with three (3+), two (2+), or one (1+) phosphorylated STAT contributing to the total response was computed.

### Gene expression analysis

A total of 2 × 10^6^ PBMCs were cultured in a 24-well plate for 24 h in AIM-V medium (Invitrogen). During the last 6 h of incubation, the cells were stimulated with the cytokine IL-27 or IL-7 at a concentration of 50 ng/mL or with the mock control containing only media. For set up, IL-27 and IL-7 were tested at 50, 100, and 200 ng/mL for 6 and 16 h [16,37]. Then, the cells were washed three times with cold PBS, and cytoplasmic RNA was isolated using the RNeasyPlus Mini Kit (Qiagen). RNA (8.5 μL) was reverse transcribed into complementary DNA (cDNA) and amplified in a total volume of 20 μL using SuperScript III First-Strand Synthesis System (ThermoFisher Scientific) with oligo(dT) primers under standard conditions. Real-time *Taq*Man polymerase chain reaction (qPCR) was performed in each sample using iQ SYBR Green Supermix (Bio-Rad) in a total volume of 20 μL following the instructions of the manufacturer. The relative gene expression of *STAT1*, *STAT3*, *STAT5*, MIG (*CXCL9*), Granzyme B (*GZMB*), T-bet (*TBX21*), and Eomesodermin (*EOMES*) was normalized to the *GADPH* expression. The sequences of the primers used are listed in Table 2. The data were analyzed with Bio-Rad CFX Manager 3.0.

**Table 2:** Primers for Real Time RT-PCR.

Gene	Primers	
*STAT1*	F:GTGCCCTGTTGAAGGACCA	R: TCAACCGCATGGAAGTCAGG
*STAT3*	F: CATCCTGAAGCTGACCCAGG	R:TATTGCTGCAGGTCGTTGGT
*STAT5B*	F: GAAGATCAAGCTGGGGCACT	R: GCTTCTCGGACCAACCTCTG
*TBX21*	F: CCACCTGTTGTGGTCCAAGT	R: CATCCTGTAGTGGCTGGTGG
*EOMES*	F:AGGTTCTGGCTTCCGTGC	R:GCAGTGGGATTGAGTCCGTT
*GZMB*	F: GCCCAGGGCAGATGCAG	R: CTCGTATCAGGAAGCCACCG
*CXCL9*	F:GTGGTGTTCTTTTCCTCTTGGG	R: AACAGCGACCCTTTCTCACT
*GADPH*	F: ACCCACTCCTCCACCTTTGAC	R:TCCACCACCCTGTTGCTGTAG

### IFN-γ enzyme-linked immunosorbent spot (ELISPOT) assays

The number of *T*. *cruzi*-specific IFN-γ-producing T cells was determined by *ex vivo* ELISPOT assays with a commercial kit (BD), as described elsewhere [14]. For set-up, IL-27 and IL-7 were tested at 25, 50, and 100 ng/mL, and the selected concentration was 50 ng/mL for both cytokines [14].

### Intracellular staining for cytokine production

To assess IL-10 production by T cells, 4 × 10^6^ PBMCs were incubated for 20 h in the presence or absence of 100 ng/mL of IL-27 [21] at 37°C in a CO_2_ incubator. For the set-up of the IL-10 production assay, IL-27 was tested at 25, 50, and 100 ng/mL. Brefeldin A (10 μg/mL; BD 554724) was added for the last 5 h of incubation, as previously described [38]. Stimulation with Staphylococcal Enterotoxin B (SEB) (1 μg/mL; Sigma Aldrich) served as a positive control. The cells were then stained with a combination of FV510, anti-CD4 PerCP and CD3 APC-Cy7 monoclonal antibodies for 30 min at 4°C followed by fixation and permeabilization (CytoFix/CytoPerm, BD) for intracellular staining with anti-IL-10 (BD).

For the polyfunctional T-cell response assays, PBMCs were incubated with 15 μg/mL of *T*. *cruzi* lysate preparation [6] or media alone plus 1 μg/mL CD28/CD49d (BD, 347690) in the presence or absence of 50 ng/mL IL-27 or IL-7, as previously described [14]. After fixation and permeabilization (CytoFix/CytoPerm, BD), the cells were stained with a combination of anti-IFN-γ, MIP-1β, CD154, TNF-α and IL-2 monoclonal antibodies as previously described [38]. For these assays, the lymphocytes were gated as described above and then analyzed for CD4 *vs*. each marker. For the analysis of cytokine coexpression profiles with one (1+), two (2+), three (3+), four (4+) or five (5+) functions, the Boolean gating function of FlowJo software was used (S2 Fig). A total of 31 different T-cell populations were obtained from the combination of the five different T-cell functions analyzed. Gating of populations was determined by the use of FMO controls. To obtain *T*. *cruzi*-specific frequencies, the background responses detected in the negative control samples containing only media or containing media along with IL-27 or IL-7 were subtracted from those detected in the *T*. *cruzi*-stimulated samples for each cytokine-producing T cell or the 31 combinations. To determine the cut-off values for responses to *T*. *cruzi* antigens, the average of *T*. *cruzi*-specific T-cell responses plus three standard deviations for three uninfected donors was calculated. The T-cell responses for each functional combination were considered positive when they were higher than the cut-off value, at least twice the response with media alone and if there were at least three events for polyfunctional responses [38,39].

To analyze the phenotype of cytokine-producing T cells, the cells were also stained for anti-PD-1 PE-Cy7 and CD57 PE-CF594 monoclonal antibodies before cell permeabilization and anti-Bcl-2 FITC after cell permeabilization. Phenotypic analysis was only performed in cytokine-producing T-cells which met the positiveness criterion for each cytokine and stimuli and had at least 20 events in each gate.

### Statistical analysis

**Fig 1:**
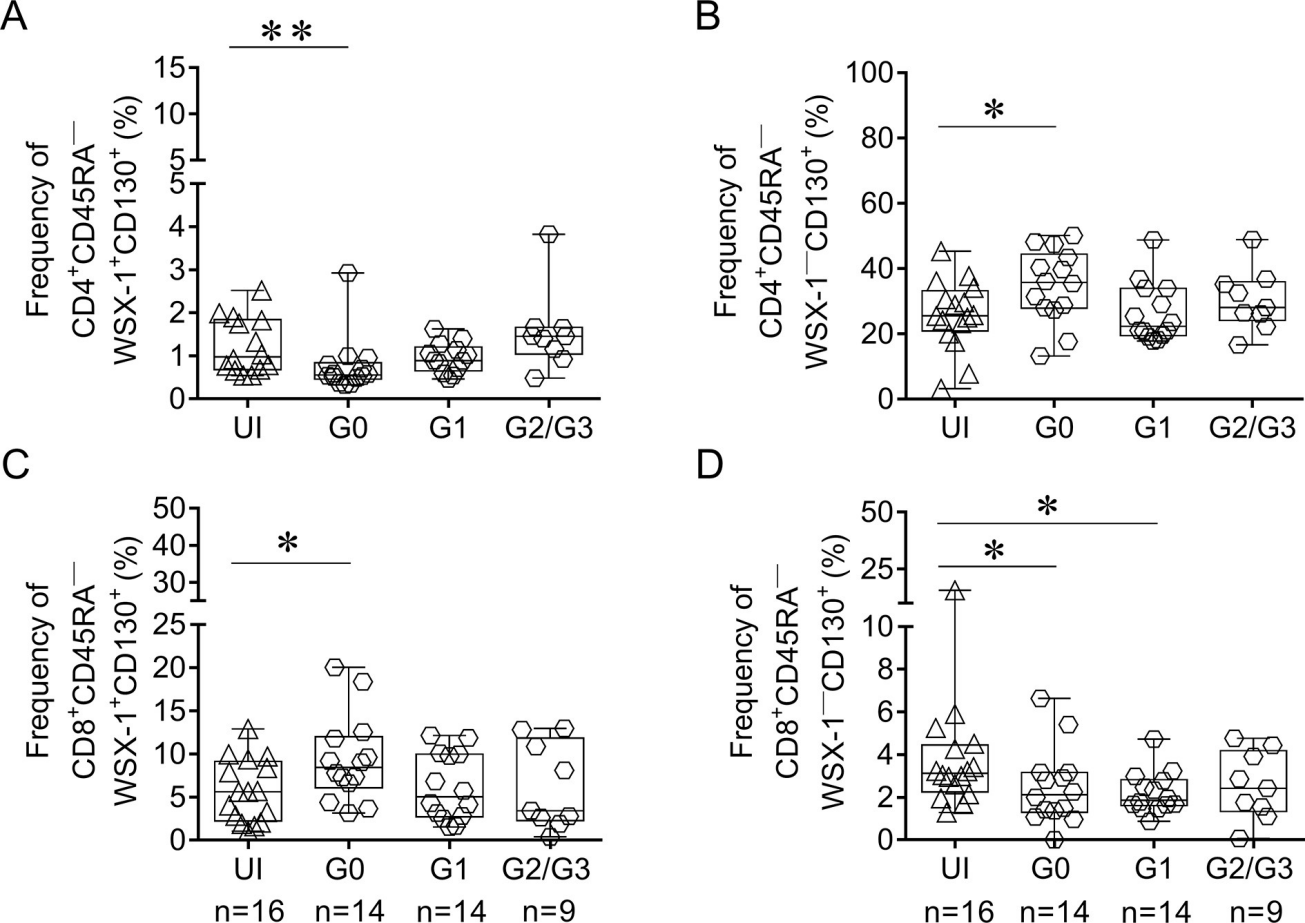
Differential expression of the IL-27R components in CD4^+^ and CD8^+^ antigen-experienced T cells in chronic *T*. *cruzi*-infected subjects with different degrees of cardiac dysfunction. PBMCs from *T*. *cruzi*-infected and uninfected subjects (UI) were stained with the dye FV510 and the monoclonal antibodies for CD3, CD4, CD8, CD45RA, WSX-1 and CD130 and analyzed using flow cytometry. Each symbol represents the frequency of antigen-experienced CD45RA^—^WSX-1^+^CD130^+^ (A, C) and CD45RA^—^WSX-1^—^CD130^+^ (B, D) CD4^+^ and CD8^+^ cells, relativized to the individual values of CD4^+^ and CD8^+^ for each subject. Medians are indicated by the horizontal lines; boxes indicate the 10–90 percentile range. *P < 0.05, ** P < 0.01 compared with UI by the Kruskal-Wallis test followed by Dunn’s test (A-B) or ANOVA followed by Bonferroni’s test (C-D), as appropriate.

The data in Figs 1, 3 and 5, and 6 were relativized according to the individual values of CD4^+^ and CD8^+^ T cells to represent the frequencies. The Shapiro-Wilk test was applied to determine the normality of data. Differences between each clinical group and the uninfected group were determined by an analysis of variance (ANOVA) followed by Bonferroni’s multiple comparisons test or the Kruskal-Wallis test followed by Dunn’s multiple comparisons test, as appropriate, or by a test for linear trend. A paired *t* test or Wilcoxon matched-pairs test was applied to determine the differences between stimulated and unstimulated cell cultures from the same donors. Only P < 0.05, along with a median increase of at least 50% between cytokine-stimulated and unstimulated cell cultures in STAT phosphorylation and Bcl-2 expression, was considered statistically significant. An unpaired t-test was used when comparing cell cultures from different donors. For neutralizing assays with IL-2, the ratio between cytokine-stimulated and unstimulated cultures in the presence or absence of neutralizing antibodies was compared using the Mann-Whitney U test.

## Results

### Differential expression of the IL-27R components in CD4^+^ and CD8^+^ T cells of subjects with chronic *T*. *cruzi* infection

The expression of the IL-27R components, WSX-1 and CD130, was evaluated in CD4^+^ and CD8^+^ antigen-experienced (CD45RA^—^, S1 Fig) T cells of subjects with different clinical forms of Chagas disease. Patients without heart disease (i.e., the G0 group) had a decreased frequency of antigen-experienced CD4^+^ T cells expressing both chains of IL-27R (Fig 1A), with a reciprocal increase in the frequency of CD4^+^CD45RA^—^WSX-1^—^CD130^+^ T cells^,^ (Fig 1B), compared with uninfected subjects. In contrast, subjects in the G0 group had an increased frequency of CD8^+^CD45RA^—^WSX-1^+^CD130^+^ T cells (Fig 1C) along with a decrease in the frequency of CD8^+^CD45RA^—^WSX-1^—^CD130^+^ T cells (Fig 1D) compared with uninfected subjects. In contrast, the expression of IL-27R components in CD4^+^ and CD8^+^ T cells was unaltered in patients with mild cardiac manifestations (G1 group) and in those with severe disease in the G2/G3 group. CD4^+^ and CD8^+^ T cells with intermediate phenotypes of the IL-27R phenotypes formed minor populations (S1 Fig) and did not exhibit significant differences among the clinical groups (P > 0.05).

### *Trypanosoma cruzi* triggers downregulation of IL-27R in CD4^+^ T cells and upregulation in CD8^+^ T cells *in vitro* in patients without cardiac disease

**Fig 2:**
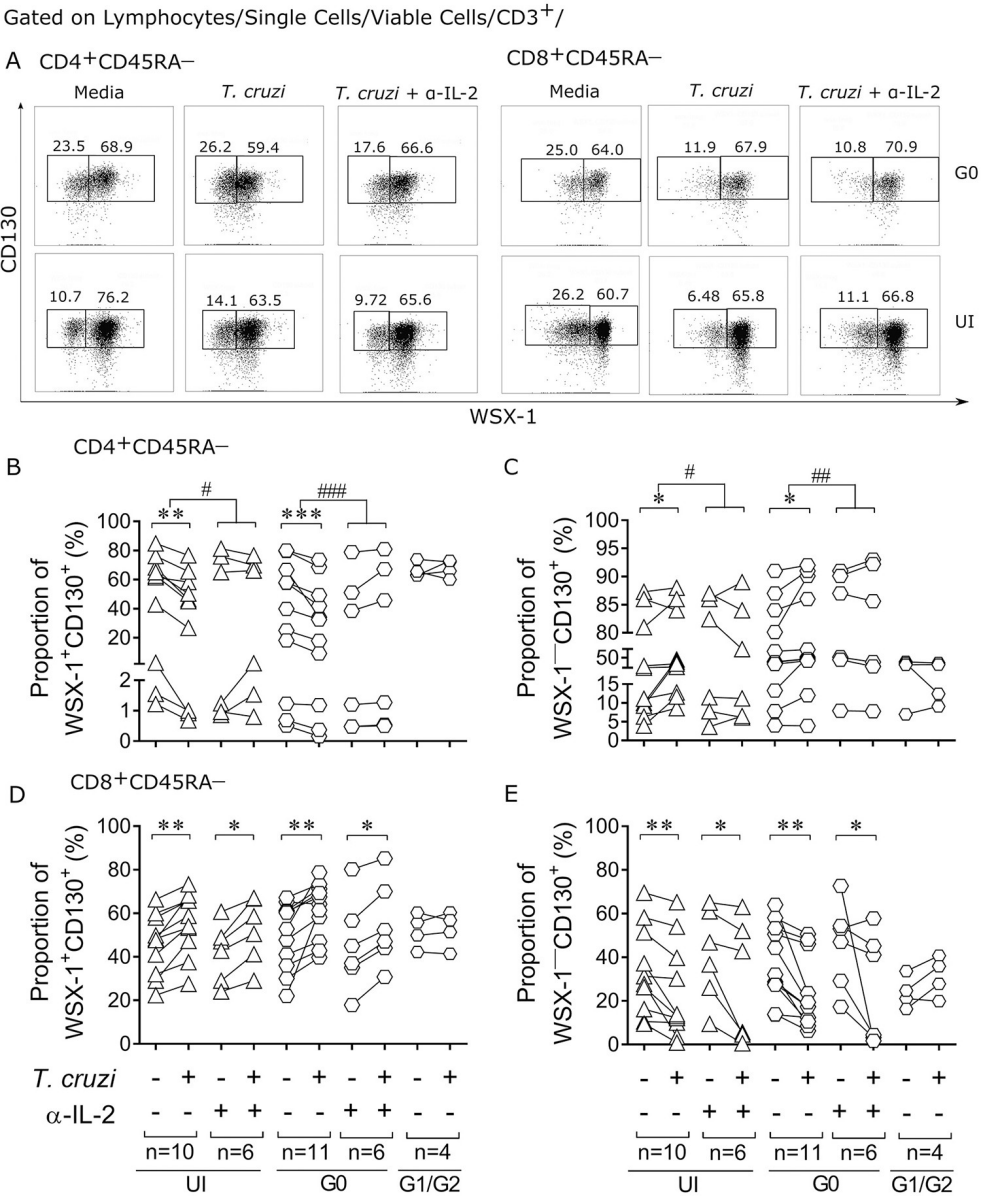
Modulation of IL-27R components after *in vitro* infection of PBMCs with *T*. *cruzi* trypomastigotes. The PBMCs of Chagas disease patients with no signs of heart disease (i.e., the G0 group) and with cardiomyopathy (i.e., the G1/G2 groups) and uninfected subjects (UI) were co-cultivated with *T*. *cruzi* trypomastigotes for 48 h. The cells were stained with the dye FV510 and the monoclonal antibodies for CD3, CD4, CD8, CD45RA, WSX1 and CD130 and analyzed using flow cytometry. Representative data of the pattern of WSX-1 and CD130 expression on CD4^+^CD45RA^—^(A, left) or CD8^+^CD45RA^—^(A, right) T cells of a G0 patient (upper panels) and an uninfected subject (lower panels). Data are shown as dot plots of basal IL-27R expression (media, left panels), IL-27R expression after coculture with *T*. *cruzi* trypomastigotes (*T*. *cruzi*, middle panels) and IL-27R expression after coculture with *T*. *cruzi* trypomastigotes and with the addition of anti-IL-2 neutralizing antibodies (*T*. *cruzi +* α-IL-2, right panels). The lower figures show the proportions of WSX-1^+^CD130^+^ (right quadrants) and WSX-1^—^CD130^+^ (left quadrants). Each symbol represents the proportions of cells expressing both IL-27R components (WSX-1^+^CD130^+^) or with downmodulated WSX-1 chain (WSX-1^—^CD130^+^) of each subject in CD45RA^—^cells in the total CD4^+^ (B-C) and CD8^+^ (D-E) T cell compartments. * P < 0.05, ** P < 0.01, and *** P < 0.001 compared with unstimulated cultures using paired *t*-test. ^#^ P < 0.05, ^##^ P < 0.01, and ^###^ P < 0.001 indicate the differences in the ratio of *T*. *cruzi*-stimulated and unstimulated cultures in the presence or absence of neutralizing IL-2 antibodies in each clinical group using the Mann Whitney *U* test.

To assess whether *T*. *cruzi* infection *in vitro* could mimic the changes in the expression of the IL-27R components observed *ex vivo*, the expression of WSX-1 and CD130 was analyzed in antigen-experienced T cells after *in vitro* exposure of PBMCs to *T*. *cruzi* trypomastigotes. *T*. *cruzi* induced a downregulation of the expression of the IL-27R in CD4^+^ T cells (Fig 2A–2C) along with an upregulation in CD8^+^ T cells of uninfected subjects and chronic Chagas disease patients with no signs of heart disease (i.e., the G0 group) (Fig 2A and 2D–2E). The ratio of CD4^+^ and CD8^+^ T cells expressing the IL-27R components before and after *T*. *cruzi* infection *in vitro* in the group of patients with heart disease symptoms (i.e., the G1/G2 group) was significantly lower than that in G0 and uninfected subjects (Fig 2A G1/G2 *vs*. UI P = 0.012, G1/G2 *vs*. G0 P = 0.005; Fig 2D G1/G2 *vs*. UI P = 0.002, G1/G2 *vs*. G0 P = 0.005).

As IL-2 is predominantly secreted by CD4^+^ T cells [40] and inhibits the expression of WSX-1 in activated CD4^+^ T cells in an autocrine manner [29], we also evaluated the effect of neutralizing IL-2 on the IL-27R expression in T cells in *in vitro* IL-27-stimulation assays. The downregulation of the IL-27R expression in CD4^+^ T cells was inhibited (Fig 2A–2C), whereas the upregulation of the IL-27R in CD8^+^ T cells was not affected by IL-2 neutralization (Fig 2A and 2D–2E). As a whole, *T*. *cruzi* induced downregulation and upregulation of IL-27R in CD4^+^ and CD8^+^ T cells, respectively, in subjects with or without mild cardiac disease, while IL-27R remained unaltered in patients with severe disease.

### IL-27-induced STATs phosphorylation is perturbed in chronic *T*. *cruzi*-infected subjects

**Fig 3:**
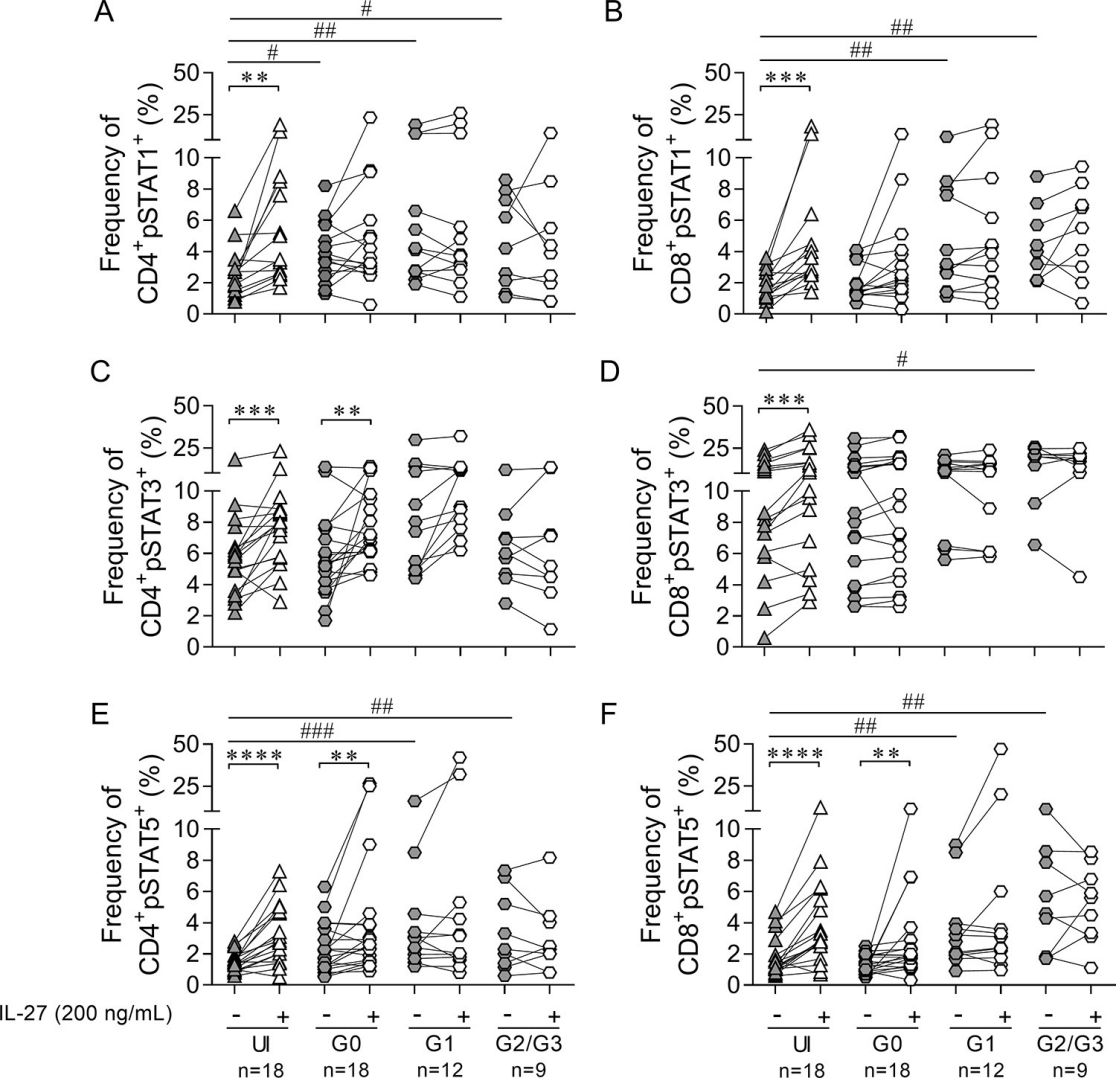
IL-27-induced phosphorylation of STAT1, STAT3, and STAT5 *in vitro* is reduced in chronic Chagas disease cardiomyopathy. Each symbol represents STAT1 (A-B), STAT3 (C-D) and STAT5 (E-F) phosphorylation before and after stimulation with IL-27 of PBMCs *in vitro* (% pSTAT^+^) in CD4^+^ (A, C, E) and CD8^+^ (B, D, F) T cells as evaluated using flow cytometry. Each symbol represents the frequency of CD4^+^/CD8^+^pSTAT1^+^, pSTAT3^+^ and pSTAT5^+^ for individual subjects, relativized to the individual values of CD4^+^ and CD8^+^ for each subject. Comparisons of the frequency of CD4^+^ and CD8^+^ T cells expressing the corresponding pSTAT in IL-27-stimulated cell cultures and that in unstimulated cell-cultures in each group (shown as * P < 0.05; ** P < 0.01, *** < 0.001 and **** P < 0.0001) were performed by paired *t* test. Only P < 0.05, along with a median increase of at least 50% in pSTAT^+^ T cells, was considered statistically significant. Comparisons of the frequency of CD4^+^ and CD8^+^ T cells expressing the corresponding pSTAT in unstimulated cell-cultures (full gray symbols, shown as ^#^ P < 0.05; ^##^ P < 0.01 and ^###^ P < 0.001) among the clinical groups and the uninfected subjects (UI) were performed by a Kruskal-Wallis test followed by Dunn’s test for multiple comparisons. There is a positive trend in the percentages of constitutive CD4^+^ or CD8^+^ T cells expressing pSTAT1 (A, P = 0.025 slope (m) = 1.29 and B, P = 0.0019 m = 1.08); pSTAT3 (C, P = 0.025 m = 1.78 and D, P = 0.032 m = 1.83) and STAT5 (F, P = 0.0001 m = 1.08) as the clinical stage becomes more severe.

**Fig 4:**
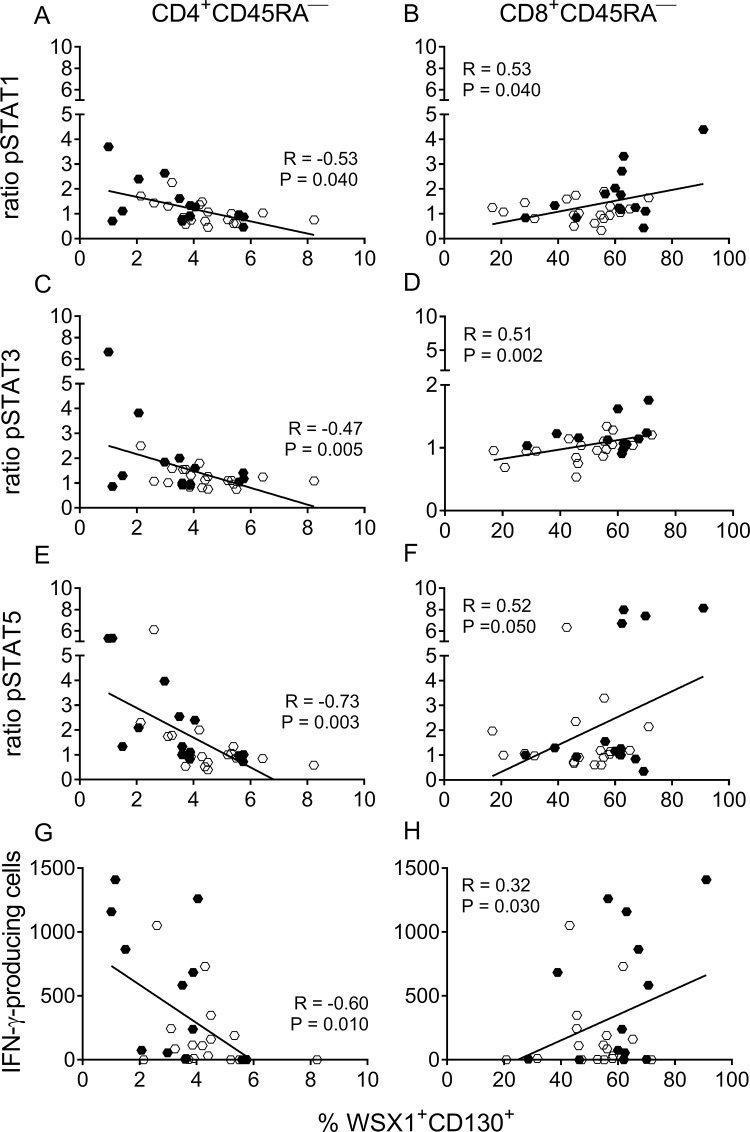
Expression of unmodulated IL-27R components associates with low STAT phosphorylation and IFN-γ production in response to *T*. *cruzi* antigens. The Spearman test was applied to assess the correlation between the frequency of memory CD4^+^CD45RA^—^(A, C and E) and CD8^+^CD45RA^—^(B, D and F) T cells expressing both chains of the IL-27R and the IL-27-induced STAT phosphorylation (post-stimulation/pre-stimulation ratio) in CD4^+^ and CD8^+^ T cells, respectively; the frequency of CD4^+^ (G) and CD8^+^ (H) T cells expressing both chains of the IL-27R and the total number of IFN-γ-producing cells in response to *T*. *cruzi* antigens. Full symbols represent values from subjects without heart disease symptoms (i.e., the G0 group); open symbols represent values from subjects with cardiac dysfunction (i.e., the G1, G2 and G3 groups).

A JAK-dependent event that occurs immediately after IL-27 binding to its receptor is phosphorylation of STAT1 (pSTAT1), STAT3 (pSTAT3), and STAT5 (pSTAT5) [41]. In response to IL-27, the frequency of total CD4^+^ and CD8^+^ T cells with phosphorylated STAT1, STAT3, and STAT5 were analyzed separately, and were observed to significantly increase in uninfected subjects (Fig 3A–3F). *Trypanosoma cruzi*-infected subjects without heart disease (i.e., G0 clinical group) also showed an increased frequency of CD4^+^ (Fig 3C and 3E) and CD8^+^ (Fig 3F) T cells while expressing pSTAT3 or pSTAT5, but not pSTAT1, in response to IL-27. However, the magnitude of phosphorylation of STAT5 in G0 subjects was lower than that in uninfected subjects (P < 0.05). In contrast, in patients with heart disease (i.e., G1, G2/G3 groups), STAT phosphorylation did not significantly increase after IL-27 stimulation. On an individual basis, the number of subjects with augmented STAT phosphorylation in response to IL-27 was significantly lower in the G2/G3 subject group than those in the uninfected group (S3 Fig). In addition, simultaneous STAT1, STAT3, and STAT5 phosphorylation in response to IL-27 decreased with disease severity (S3 Fig). The frequency of CD4^+^ T cells expressing IL-27R was inversely correlated with the levels of pSTAT1, pSTAT3, and pSTAT5 (Fig 4A–4F), whereas a positive association was observed between the frequency of CD8^+^ T cells expressing IL-27R and the levels of phosphorylated STATs (Fig 4A–4F), supporting the differential regulation of IL-27R in CD4^+^ and CD8^+^ T cells. Low levels of STAT phosphorylation in response to IL-27 in subjects with heart disease were associated with increased constitutive STAT phosphorylation (Fig 3) and increased constitutive gene expression of these transcription factors (S4A–S4C Fig). STAT3 constitutive phosphorylation was higher in CD8^+^ T cells than that in CD4^+^ T cells at all clinical stages (P < 0.05), which was associated with a lower IL-27-induced phosphorylation in CD8^+^ T cells than that in CD4^+^ T cells (P < 0.05). Overall, downregulation of IL-27R in CD4^+^ T cells and upregulation in CD8^+^ T cells reflected a functional IL-27R axis with active STAT phosphorylation after IL-27 stimulation, whereas increased constitutive phosphorylated STAT was associated with unresponsiveness to IL-27.

### Downstream STATs gene expression is altered in patients with cardiomyopathy and chronic *T*. *cruzi* infection

In response to IL-27, gene expression of *TBX21*, *EOMES*, and *CXCL9*, which are activated downstream of STAT1, STAT3, and STAT5 phosphorylation, was lower in patients with cardiomyopathy (i.e., the G1 and G2 groups) than that in uninfected subjects (S4D–S4E and S4G Fig). Of note, although not statistically significant, *EOMES* expression was also low in some patients in the G0 subject group (S4E Fig), whereas no changes were observed in *GZMB* expression (S4F Fig). As IL-27 and IL-7 share signaling through these STATs [42,43], the fold change of these transcription factors was also evaluated after IL-7 stimulation. Patients with cardiomyopathy showed decreased expression of *TBX21*, *EOMES*, and *CXCL9* (S4H–S4SI and S4K Fig) and unaltered *GZMB* (S4J Fig), while no difference was observed in *T*. *cruzi*-infected subjects without heart disease (S4H–S4K Fig) compared with uninfected subjects.

### IL-27 and IL-7 improve *T*. *cruzi*-responsive polyfunctional T cells

**Fig 5:**
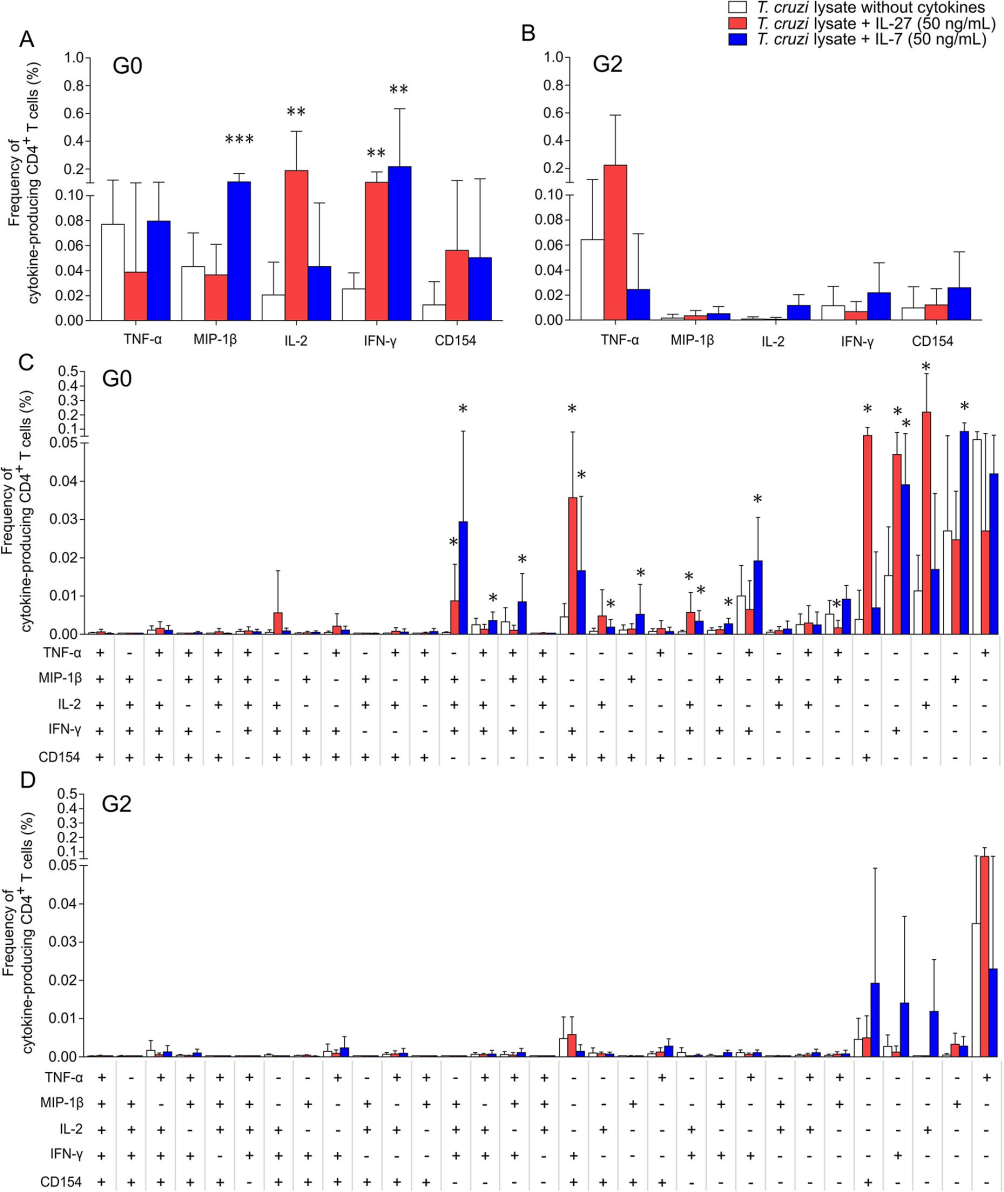
Polyfunctionality of *T*. *cruzi*-specific CD4^+^ T cells in patients with chronic Chagas disease with no sign of cardiomyopathy is improved after *in vitro* treatment with IL-27 and IL-7. PBMCs of chronic *T*. *cruzi*-infected subjects with no signs of heart disease (n = 10) and subjects with heart disease (i.e., the G2 group, n = 4) were stimulated with a *T*. *cruzi* lysate preparation from the Brazil strain in the presence or absence of IL-27 (red bars) or IL-7 (blue bars) and analyzed using flow cytometry for the intracellular expression of TNF-α, MIP-1β, IL-2, IFN-γ and CD154 in CD4^+^ T cells. Each bar represents the frequency of total TNF-α-, MIP-1β-, IL-2-, IFN-γ- and CD154-*T*. *cruzi*-specific CD3^+^CD4^+^ T-cell responses (A and B). For the analysis of polyfunctional T-cell responses, the cytokine coexpression profiles with one (1+), two (2+), three (3+), four (4+) and five (5+) functions were determined using the Boolean gating function of FlowJo software. Each bar represents the total frequency of *T*. *cruzi*-specific (i.e., values obtained in cultures with only media were subtracted) CD3^+^CD4^+^ T-cell responses of each cytokine-producing subset relative to the individual values of CD4^+^ for each subject (C and D). * P < 0.05, ** P < 0.01, *** P < 0.001 compared with *T*. *cruzi*-specific T-cell responses without the addition of cytokine (white bars) according to a paired *t* test. Data are shown as the means and SD.

We then evaluated whether the addition of IL-27 or IL-7 might improve CD4^+^ T cell polyfunctional response to *T*. *cruzi* antigens, considering the simultaneous production or expression of molecules mediating Th1 responses. Examining TNF-α, MIP-1β, IL-2, IFN-γ and CD154 individually, the addition of IL-27 increased the frequency of IL-2 and IFN-γ-producing CD4^+^ T cells in response to *T*. *cruzi* antigens, while IL-7 induced the production of MIP-1β and IFN-γ in subjects of the G0 group (Fig 5A). Analysis of the frequencies of *T*. *cruzi*-specific T cells with every possible combination of the five T-cell functions using the Boolean gating tool showed that in subjects without signs of heart disease (i.e., the G0 group), IL-27 mainly increased the frequencies of monofunctional IFN-γ-, IL-2-, and CD154-producing CD4^+^ T cells, whereas IL-7 increased the frequencies of monofunctional IFN-γ^+^ and MIP-1β^+^ CD4^+^ T cells (Figs 5C and S2). Among polyfunctional T cells, both IL-27 and IL-7 increased the frequencies of T cells with three and two functions, mostly expressing IL-2, IFN-γ, MIP-1β, and CD154 (Fig 5C). In contrast to that observed in subjects without cardiomyopathy, the addition of IL-27 or IL-7 in *T*. *cruzi*-stimulated cell cultures of PBMCs derived from those with heart disease (i.e., the G2 group) did not improve the frequencies of monofunctional or polyfunctional CD4^+^ T-cell responses specific for *T*. *cruzi*. (Fig 5B and 5D). Although not statistically significant, the frequency of TNF-α^+^ cells in single cytokine-producing T cells increased after *in vitro* treatment with IL-27 (Fig 5B and 5D). *In vitro* culture of PBMCs from uninfected subjects with the addition of IL-27 and IL-7 did not change the frequency of cytokine-producing CD4^+^ T cells in response to *T*. *cruzi* antigens (S5 Fig).

**Fig 6:**
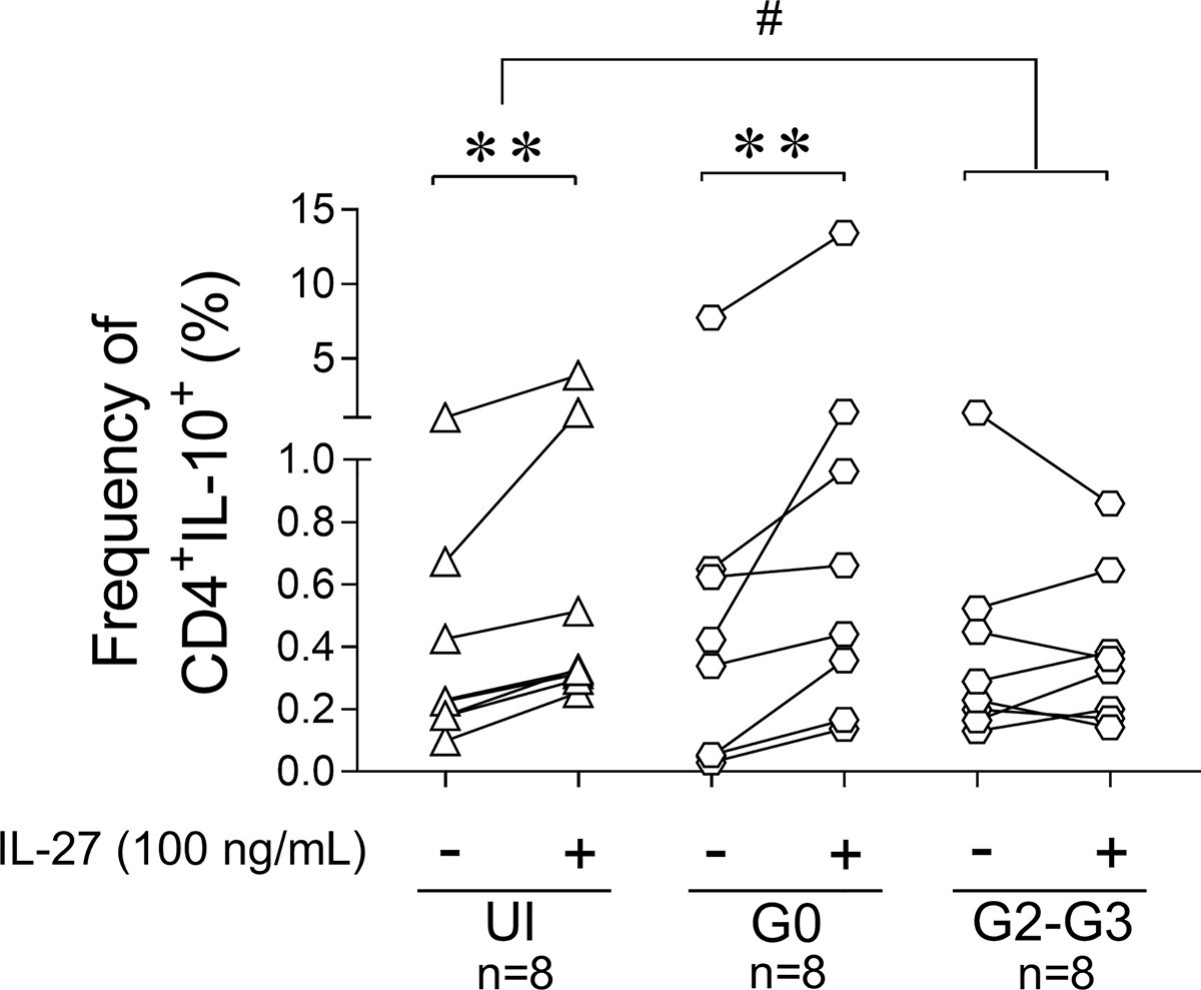
IL-10 production in response to IL-27 is impaired in Chagas disease patients with severe cardiomyopathy. PBMCs of subjects with chronic Chagas disease with no signs of heart disease (i.e., the G0 group) or with myocardiopathy (i.e., the G2-G3 group) and of uninfected subjects (i.e., UI) were stimulated with 100 ng/mL IL-27 for 20 h and then evaluated for IL-10 production in CD4^+^ T cells using flow cytometry. Each point represents the frequency of CD4^+^IL-10^+^ T cells, relativized to the individual values of CD4^+^ and CD3^+^ for each subject. * P <0.05 compared with unstimulated cell culture according to a paired *t* test.^.#^ P < 0.05 between post-stimulation/pre-stimulation ratios of the indicated subject groups. Only P < 0.05, along with a median increase of at least 50% in IL-10^+^ T cells, was considered statistically significant.

The low production of polyfunctional T cells in patients with severe cardiomyopathy was not due to enhanced IL-27-induced IL-10 production [44], but in contrast, IL-10 was only induced in CD4^+^ T cells of the G0 subject group and in uninfected subjects (Figs 6 and S6). Using the ELISPOT technique, we observed that the total number of IFN-γ-producing cells in response to *T*. *cruzi* without the addition of cytokines was inversely associated with the frequency of memory CD4^+^CD45RA^—^T cells expressing both chains of IL-27R (i.e., WSX-1^+^CD130^+^) and positively associated with the frequency of memory CD8^+^ CD45RA^—^WSX-1^+^CD130^+^ (Fig 4G–4H), further supporting that modulation of IL-27R is associated with parasite-specific T-cell responses.

### IL-27 and IL-7 increase Bcl-2 expression by *T*. *cruzi*-responsive CD4^+^ T cells

**Fig 7:**
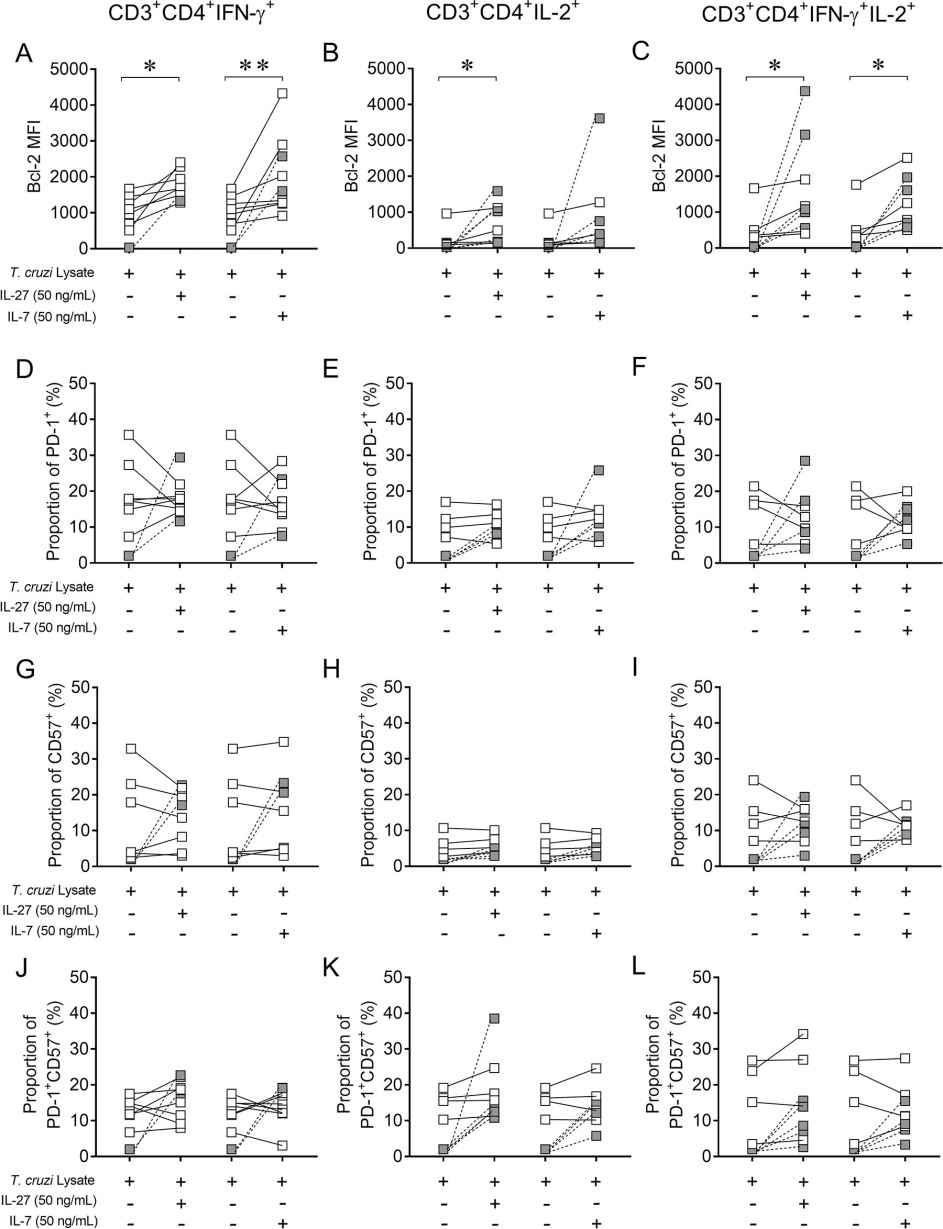
Bcl-2 expression is higher in IFN-γ- and IL-2-coproducing T cells in response to *T*. *cruzi* antigens after treatment with IL-27 and IL-7. PBMCs of chronic *T*. *cruzi*-infected subjects with no signs of heart disease (n = 10) were stimulated with a *T*. *cruzi* lysate preparation from the Brazil strain in the presence or absence of IL-27 or IL-7. Flow cytometry analysis was applied to determine the expression of Bcl-2 (A-C), PD-1^+^ (D-F), and CD57^+^ (G-I) or the coexpression of PD-1 and CD57 (J-L) cells in IFN-γ- and IL-2-producing T cells. Each different symbol represents data for a specific patient. IL-27 or IL-7-stimulated and unstimulated samples are bound by lines. Gray symbols bound by dotted lines represent data of subjects in whom *T*. *cruzi*-specific cytokine-producing T cells in response to *T*. *cruzi* lysate were undetectable in the absence of IL-27 or IL-7 stimulation, which became detecs upon IL-27 or IL-7 stimulation. Data were analyzed by a paired *t* test. * P < 0.05; ** P < 0.01 compared with *T*. *cruzi* stimulated cell cultures without the addition of cytokines. Only P < 0.05, along with a median increase of at least 50% in cytokine-producing cells expressing Bcl-2, PD-1 or CD57, was considered statistically significant.

We further explored the phenotypic characteristics of IFN-γ- and IL-2-producing CD4^+^ T cells in response to *T*. *cruzi* antigens that emerged after *in vitro* treatment with IL-27 and IL-7. Stimulation with IL-27 and IL-7 significantly increased Bcl-2 expression in individual IFN-γ^+^- and dual IFN-γ^+^IL-2^+^CD4^+^ T cells compared to that in cell cultures not stimulated with cytokines (Fig 7A–7C), whereas only IL-27 induced Bcl-2 expression in individual IL-2^+^CD4^+^ T cells (Fig 7B). In certain subjects, in whom cytokine-producing T cells formed in response to stimulation with the *T*. *cruzi* lysate were undetectable, CD4^+^ T cells with individual or dual function and expressing Bcl-2 became detectable after stimulation with *T*. *cruzi* antigens and IL-27 or IL-7 (Fig 7A–7C, gray symbols). In contrast, no changes were observed in the expression of the inhibitory receptors PD-1 and CD57 in IFN-γ- or IL-2-producing CD4^+^ T cells (Fig 7D–7L).

## Discussion

Currently, as is in chronic viral infections, chronic parasitic infections can exhaust adaptive immunity [45]. Although several studies have shown functionally defective T-cell phenotypes with poor recall/memory abilities to *T*. *cruzi* antigens, little is known about the cell-intrinsic mechanisms of T-cell exhaustion in chronic Chagas disease [6,10,46–50]. Herein, we showed that the function and expression of the IL-27 receptor and the gene expression of transcription factors involved in IL-27 signaling pathways were altered in *T*. *cruzi*-infected subjects presenting cardiac disease, and CD4^+^ T-cell responses to *T*. *cruzi* were improved upon the exogenous addition of IL-27 and IL-7 only in the face of functional IL-27/IL-27R and IL-7/IL-7R pathways. The increase in the number of *T*. *cruzi*-specific CD4^+^ T cells was associated with an increase in the number of IL-10-producing CD4^+^ T cells after IL-27 stimulation. These observations are in agreement with the ability of IL-27 to promote pro-inflammatory and anti-inflammatory functions [27], as well as to regulate the Th1 response in patients without cardiac disease to keep the parasite under control without tissue damage [13,32]. Several studies have also shown a low expression of IL-10 produced by PBMCs from patients with cardiac Chagas disease, reflecting an imbalanced immune response during persistent infection [11,51–53].

Our data presented here suggest a differential modulation of the expression of IL-27R between CD4^+^ and CD8^+^ T cells in the memory T-cell compartment during *T*. *cruzi* infection *in vitro* and *ex vivo*, which was associated with a milder cardiac disease. The *in vitro* assay with IL-2 blocking antibodies indicates that these findings may be explained by the inhibition of IL-27R expression by autocrine IL-2 regulation [29], a cytokine predominantly produced by CD4^+^ rather than that by CD8^+^ T cells upon activation [40]. Signaling through IL-27R during activation may also induce CD4^+^ T regulatory cell subsets to produce IL-10, which inhibits macrophages and dendritic cells, abrogates CD4^+^ T-cell activation, and further downregulates IL-27R expression [54]. In fact, CD4^+^ T cells produce IL-10 in response to IL-27 in subjects with mild disease.

During activation, a functional IL-27/IL-27R axis is reflected by the downregulation of IL-27R in CD4^+^ T cells and an upregulation in CD8^+^ T cells. *Trypanosoma cruzi*-infected subjects without heart disease also showed decreased IL-27 levels in their circulation [14], further supporting the idea that IL-27 is consumed in a functional IL-27R axis. The failure to achieve the downregulation of IL-27R in CD4^+^ or upregulation in CD8^+^ T cells in more severe stages of the disease was associated with deficient signaling through STAT and downstream genes, as well as with lower production of IFN-γ, which is a key cytokine for *T*. *cruzi* immunity [5]. The expression of the cytokine receptor without functional capacity is known as “desensitization”, and is regarded as one of the mechanisms of alteration of T-cell signaling in chronic infections [55–57]. A desensitization mechanism for several cytokines, including IL-7 and IL-6, has been described in chronic infections [56,57] and inflammation [55]. In the experimental model of T-cell exhaustion induced by infection with Lymphocytic Choriomeningitis Virus, exhausted CD4^+^ T cells are subjected to ongoing chronic STAT1 activation and a desensitization of IFN-γ pathway [56]. We have shown that a desensitization mechanism of IL-7R may occur in chronic Chagas disease [14,35], and the present study supports a similar desensitization mechanism of the IL-27R pathway.

The persistent stimulation of the immune system in subjects with chronic *T*. *cruzi* infection may induce continuous activation of IL-27/IL-27R and IL-7/IL-7R, which could lead to increased constitutive phosphorylation and gene expression of different STAT molecules, particularly observed in patients with advanced cardiomyopathy, accounting for the low degree of STAT phosphorylation in response to IL-27 and IL-7 [14,35] in these subjects. The increased constitutive levels of CD4^+^pSTAT1^+^, CD4^+^pSTAT3^+^, and CD4^+^pSTAT5^+^ or the increased constitutive gene expression of these transcription factors compensated for the signal transduction defects found in cardiomyopathy patients; this was supported by the decreased gene expression of downstream STAT genes coding for T-bet (*TBX21*) and eomesodermin (*EOMES*), which are expressed in T cells and mediate T-cell differentiation and several T-cell functions, in response to IL-27 [58]. The gene expression of *CXCL9*, coding for MIG in monocytes [59], was also low in patients with cardiomyopathy, suggesting that IL-27/IL-27R and IL-7/IL-7R may be altered in other cell types.

Since several cytokines share signaling via STAT or JAK tyrosine kinases [60], perturbations in the IL-7/IL-7R and IL-27/IL-27R axis might also be induced by cytokines other than IL-7 and IL-27. Activation through the T-cell receptor can also lead to modulation of IL-27R, independent of cytokine production, which may further desensitize the IL-27/IL-7 axis [15].

T cells in subjects with a functional IL-7/IL-7R and IL-27/IL-27R axis also showed the ability to increase cytokine-producing CD4^+^ T cells and gene expression associated with the Th1 profile in response to IL-27. Of note, *T*. *cruzi*-responsive-IL-2-producing CD4^+^ T cells, which are observed in a low proportion of subjects with chronic Chagas disease [6,61], improved after stimulation with IL-27 and IL-7. Accordingly, in these subjects, the gene expression of T-bet (i.e., a mediator of Th1 differentiation and Th2 suppression) and eomesodermin (a mediator of the differentiation of effector CD8^+^ T cells) was similar to that in uninfected subjects. This is also in agreement with the higher frequencies of polyfunctional Th1-biased CD4^+^ and CD8^+^ T cells specific for *T*. *cruzi* observed in subjects with milder forms of chronic Chagas disease than in those with more severe forms [6–8]. A recent study demonstrated that IL-27 is an upstream regulator of polyfunctional CD4^+^ T-cell responses to pathogens [62]. Stimulation of PBMCs with IL-27 also induced gene expression of transcription factors that mediate T-cell migration [59].

The loss of parasite-specific polyfunctional T-cell responses, which are not only capable of a broader repertoire of functions, but also produce a nearly 10-fold higher level of cytokines on a per-cell basis than that do monofunctional CD4^+^ T cells [63], might give rise to a compensatory, less efficient inflammatory response that is able to keep the parasite under control, but at the expense of inducing tissue damage. Although a cause-effect link between T-cell exhaustion and disease progression cannot be ascertained, they are likely to be interconnected. Adult subjects, even in the G0 group, displayed signs of T-cell exhaustion, including lower frequency and magnitude of polyfunctional T cells compared with *T*. *cruzi*-infected children [7], expression of inhibitory receptors on *T*. *cruzi*-specific T cells [14,64] and total T cells [8,9], and high levels of terminal, differentiated, and apoptotic T cells [65]; all of which worsen in more severe clinical stages. CD4^+^ T cell responses improved after *in vitro* treatment with IL-27 and IL-7 only in subjects without signs of heart disease who have a more functional IL-27 and IL-7 axis and less exhausted immune system compared with those in patients with severe cardiomyopathy. Importantly, IL-2 production, which plays an important role in the proliferation and survival of Th1 cells [66], increased following *in vitro* treatment with IL-27 and IL-7.

The recovery of co-stimulation properties through CD154 expression is important for the development of Th1 cells because it activates dendritic cells and promotes cytokine production by CD4^+^ T cells [67], whereas the capacity to produce IFN-γ and TNF-α is critical for *T*. *cruzi* immunity [5,13]. In addition, the expression of Bcl-2, a molecule involved in the rescue of apoptotic cells, also increased, supporting an improvement in the survival capabilities of T cells that arose after cytokine stimulation. It has been shown that IL-27 also inhibits cleaved caspase-3 expression and downregulates the proapoptotic protein Bax through activation of STAT3 [68]. Notably, not only were the polyfunctional CD4^+^ T cells not improved following *in vitro* treatment with IL-27 and IL-7 in cardiomyopathy patients, but a skewed enrichment in TNF-α-producing CD4^+^ T cells in response to *T*. *cruzi* was also observed, which has been associated with more severe stages of chronic Chagas disease [69].

One limitation to this study is that it was not possible to ascertain whether the impairment of the IL-27R axis is a cause or a consequence of disease progression and, since *T*. *cruzi* lysate mainly induces CD4^+^ T-cell responses, information regarding CD8^+^ T-cell responses after *in vitro* cytokine treatment is lacking.

Altogether, the work presented here supports a possible dual role of the IL-27R signaling pathway in promoting *T*. *cruzi-*specific T-cell responses while limiting T-cell responses by inducing IL-10 production. Improvement in the frequency of parasite-specific T-cells was only achieved in the face of a functional cytokine signaling pathway when the T-cell compartment was not deeply exhausted. These findings contribute to a better understanding of the immune mechanisms underlying the anti-*T*. *cruzi* response in human subjects, which may be useful for developing vaccines and new therapeutic interventions.

## Supporting information

S1 Fig Gating strategy and fluorescence minus one control for WSX-1 and CD130. PBMCs were stained for FV510, CD3/CD4/CD8, CD45RA, CD130, and WSX-1 and analyzed using flow cytometry. Lymphocytes were gated based on forward scatter and side scatter parameters, followed by forward scatter area *vs*. forward scatter height parameters and side scatter area *vs*. side scatter weight for doublet discrimination. The subsequent analyses were performed on viable cells (FV510^—^) and CD3^+^ T cells. According to CD45RA expression in CD4^+^ or CD8^+^ T cells, antigen-experienced (CD45RA^—^) T cells were gated. WSX-1^+^CD130^+^ T cells were determined according to fluorescence minus one (FMO) control for CD130 (left panel) and WSX-1 (right panel). The numbers in each dot plot indicate the percentage of the gated T-cell population.
(TIF)

S2 Fig Representative Boolean gating strategy for the analysis of the cytokine co-expression profiles of CD4+ T cells after stimulation with T. cruzi antigens. The lymphocytes were gated as described in S1 Fig and then analyzed for CD4 *vs*. each cytokine. The five populations were selected, and the Boolean gating function of Flow Jo software (Tree Star) was applied to generate data for 31 different cytokine-producing T-cell populations (lower panels). The upper panels show representative dot plots for CD154, IL-2, IFN-γ, MIP-1β, and TNF-α in the CD4^+^ T cells of the FMO controls, of samples stimulated with the *T*. *cruzi* lysate without the addition of cytokines and of samples stimulated with the *T*. *cruzi* lysate with the addition of IL-27 or IL-7 at a final concentration of 50 ng/mL, as described in the Materials and Methods.
(TIF)

S3 Fig Simultaneous phosphorylation of STAT1, STAT3 and STAT5 in response to IL-27 decreased with disease severity. PBMCs were stimulated with IL-27 and analyzed for STAT1, STAT3, and STAT5 phosphorylation in CD4^+^ (upper panels) and CD8^+^ T cells (lower panels). The Boolean gating function in FlowJo software was used to determine the proportion of T cells with three (3+), two (2+), or one (1+) phosphorylated STATs. IL-27-induced phosphorylation was considered positive when the ratio of stimulated/unstimulated was > 50%. The proportion of each subset with three, two, or one phosphorylated STAT contributing to the total response was calculated. The average for each combination was assessed for all subjects in the group, and the data were summarized in pie charts, where each slice of the pie represents the fraction of the total response that consists of CD4^+^ or CD8^+^ T cells positive for one to three phosphorylated STATs (violet, green, and red, respectively). * P < 0.05, compared with those of uninfected subjects (UI) using Fisher’s exact test.
(TIF)

S4 Fig STAT constitutive gene expression and cytokine-induced gene expression of IL-27 and IL-7-mediated functions are altered in chronic Chagas disease. PBMCs were incubated for 24 h in AIM-V medium and subsequently subjected to 6 h of incubation in the presence or absence of 50 ng/mL IL-27 (D-G) or IL-7 (H-K). The total RNA was then isolated, cDNA was synthesized, and quantitative PCR was performed in all samples. Each symbol represents the constitutive gene expression of *STAT1* (A), *STAT3* (B) and *STAT5* (C) or relative gene expression in unstimulated cells or after IL-27 or IL-7 *in vitro* stimulation of *TBX21* (D, H), *EOMES* (E, I), *GZMB* (F, J), and *CXCL9* (G, K), previously normalized to *GADPH* expression. Full black symbols represent data from subjects in the G1 clinical group. Medians are indicated by the horizontal lines; boxes indicate the 10–90 percentile range. * P < 0.05, ** P < 0.01 compared with uninfected subjects (UI) by Mann-Whitney test (A-C) **#** P < 0.05 show the difference between the ratio of cytokine-stimulated and unstimulated cultures by Mann-Whitney test.
(TIF)

S5 Fig No increase in the frequency of polyfunctional CD4^+^ T cells in uninfected subjects after in vitro treatment with IL-27 and IL-7. PBMCs of uninfected subjects (i.e., the UI group, n = 3) were stimulated with *T*. *cruzi* lysate preparation from the Brazil strain in the presence or absence of IL-27 (red bars) or IL-7 (blue bars) and analyzed using flow cytometry for the intracellular expression of TNF-α, MIP-1β, IL-2, IFN-γ and CD154 in CD4^+^ T cells. The cytokine coexpression profiles with one (1+), two (2+), three (3+), four (4+) and five (5+) functions were determined using the Boolean gating function of FlowJo software. Each bar represents the frequency of *T*. *cruzi*-specific (i.e., values obtained in cultures with only media were subtracted) CD3^+^CD4^+^ T-cell responses of each cytokine-producing population relative to the individual values of CD3^+^CD4^+^ for each subject. Data are shown as the mean and SD.
(TIF)

S6 Fig IL-10 secretion in response to IL-27 stimulation is impaired in subjects with cardiac disease. PBMCs collected from subjects with chronic Chagas disease with no signs of cardiac disease (i.e., the G0 group) or with myocardiopathy (i.e., the G3 group) were stimulated with 100 ng/mL of IL-27 or with media alone for 20 h, following which IL-10 production by CD4^+^ T cells was evaluated using flow cytometry. Representative dot plots for a G0 subject (upper panels) and a G3 subject (lower panels) are shown. IL-10^+^CD4^+^ T cells were gated according to FMO controls (upper left panel).
(TIF)
