# Supplementary figures and images for: Immune exhaustion in chronic Chagas disease: Pro-inflammatory and immunomodulatory action of IL-27 in vitro
Source: PLoS Negl Trop Dis. 2021 Jun 1;15(6):e0009473. doi: 10.1371/journal.pntd.0009473 (PMC8195349; doi:10.1371/journal.pntd.0009473)

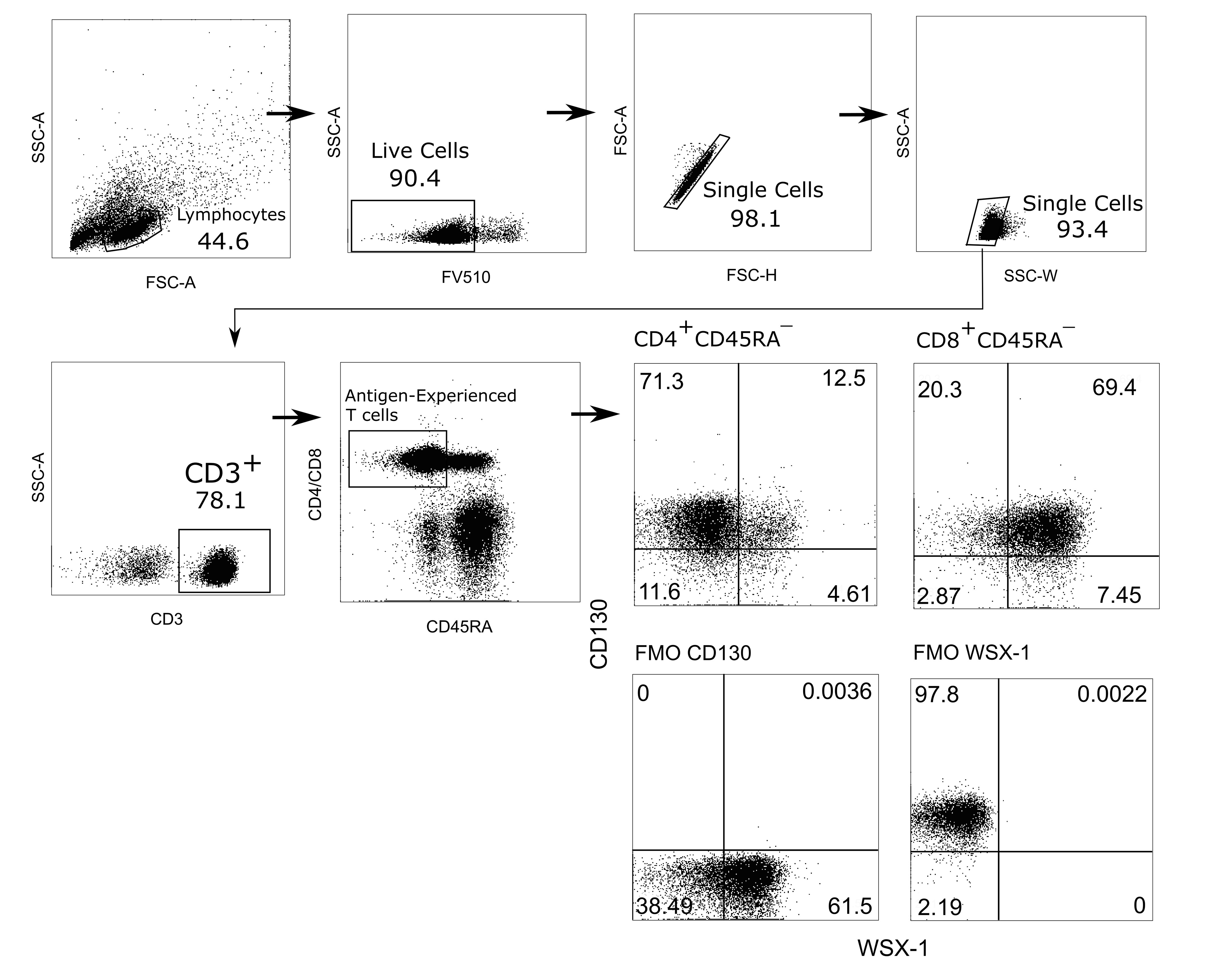

Supplement: S1 Fig — PBMCs were stained for FV510, CD3/CD4/CD8, CD45RA, CD130, and WSX-1 and analyzed using flow cytometry. Lymphocytes were gated based on forward scatter and side scatter parameters, followed by forward scatter area vs. forward scatter height parameters and side scatter area vs. side scatter weight for doublet discrimination. The subsequent analyses were performed on viable cells (FV510—) and CD3+ T cells. According to CD45RA expression in CD4+ or CD8+ T cells, antigen-experienced (CD45RA—) T cells were gated. WSX-1+CD130+ T cells were determined according to fluorescence minus one (FMO) control for CD130 (left panel) and WSX-1 (right panel). The numbers in each dot plot indicate the percentage of the gated T-cell population. (TIF) [file pntd.0009473.s001.tif]

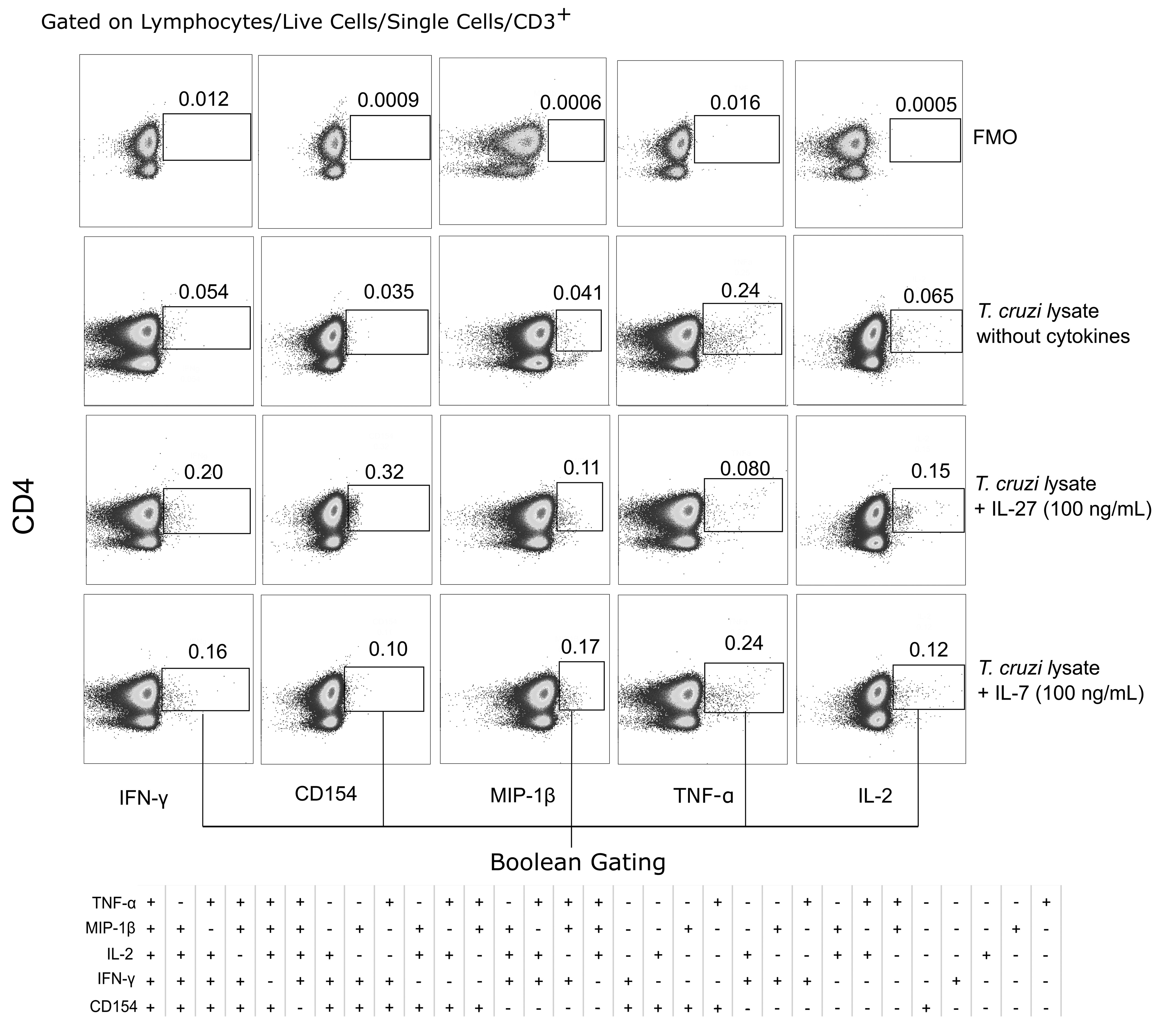

Supplement: S2 Fig — The lymphocytes were gated as described in S1 Fig and then analyzed for CD4 vs. each cytokine. The five populations were selected, and the Boolean gating function of Flow Jo software (Tree Star) was applied to generate data for 31 different cytokine-producing T-cell populations (lower panels). The upper panels show representative dot plots for CD154, IL-2, IFN-γ, MIP-1β, and TNF-α in the CD4+ T cells of the FMO controls, of samples stimulated with the T. cruzi lysate without the addition of cytokines and of samples stimulated with the T. cruzi lysate with the addition of IL-27 or IL-7 at a final concentration of 50 ng/mL, as described in the Materials and Methods. (TIF) [file pntd.0009473.s002.tif]

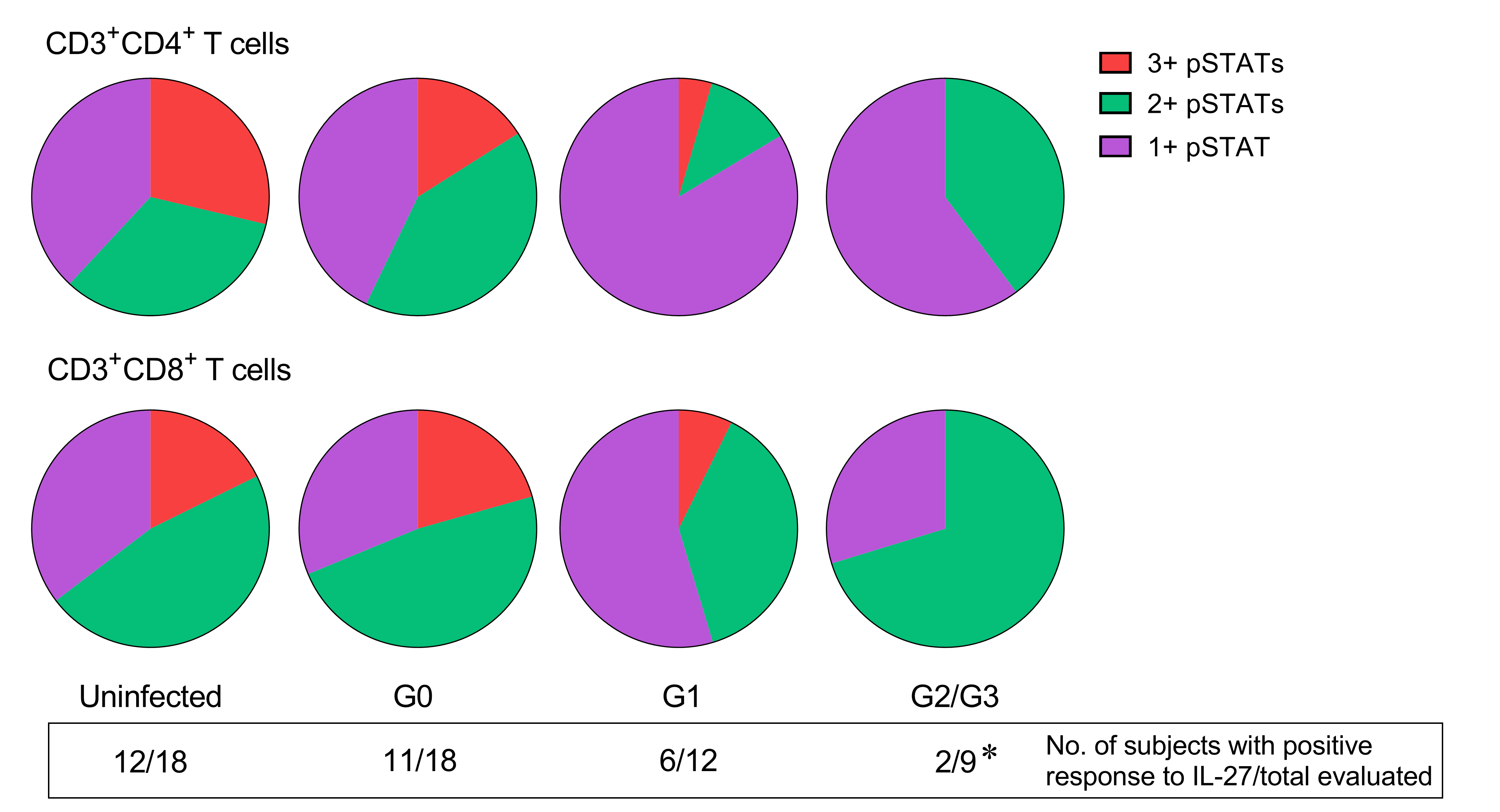

Supplement: S3 Fig — PBMCs were stimulated with IL-27 and analyzed for STAT1, STAT3, and STAT5 phosphorylation in CD4+ (upper panels) and CD8+ T cells (lower panels). The Boolean gating function in FlowJo software was used to determine the proportion of T cells with three (3+), two (2+), or one (1+) phosphorylated STATs. IL-27-induced phosphorylation was considered positive when the ratio of stimulated/unstimulated was > 50%. The proportion of each subset with three, two, or one phosphorylated STAT contributing to the total response was calculated. The average for each combination was assessed for all subjects in the group, and the data were summarized in pie charts, where each slice of the pie represents the fraction of the total response that consists of CD4+ or CD8+ T cells positive for one to three phosphorylated STATs (violet, green, and red, respectively). * P < 0.05, compared with those of uninfected subjects (UI) using Fisher’s exact test. (TIF) [file pntd.0009473.s003.tif]

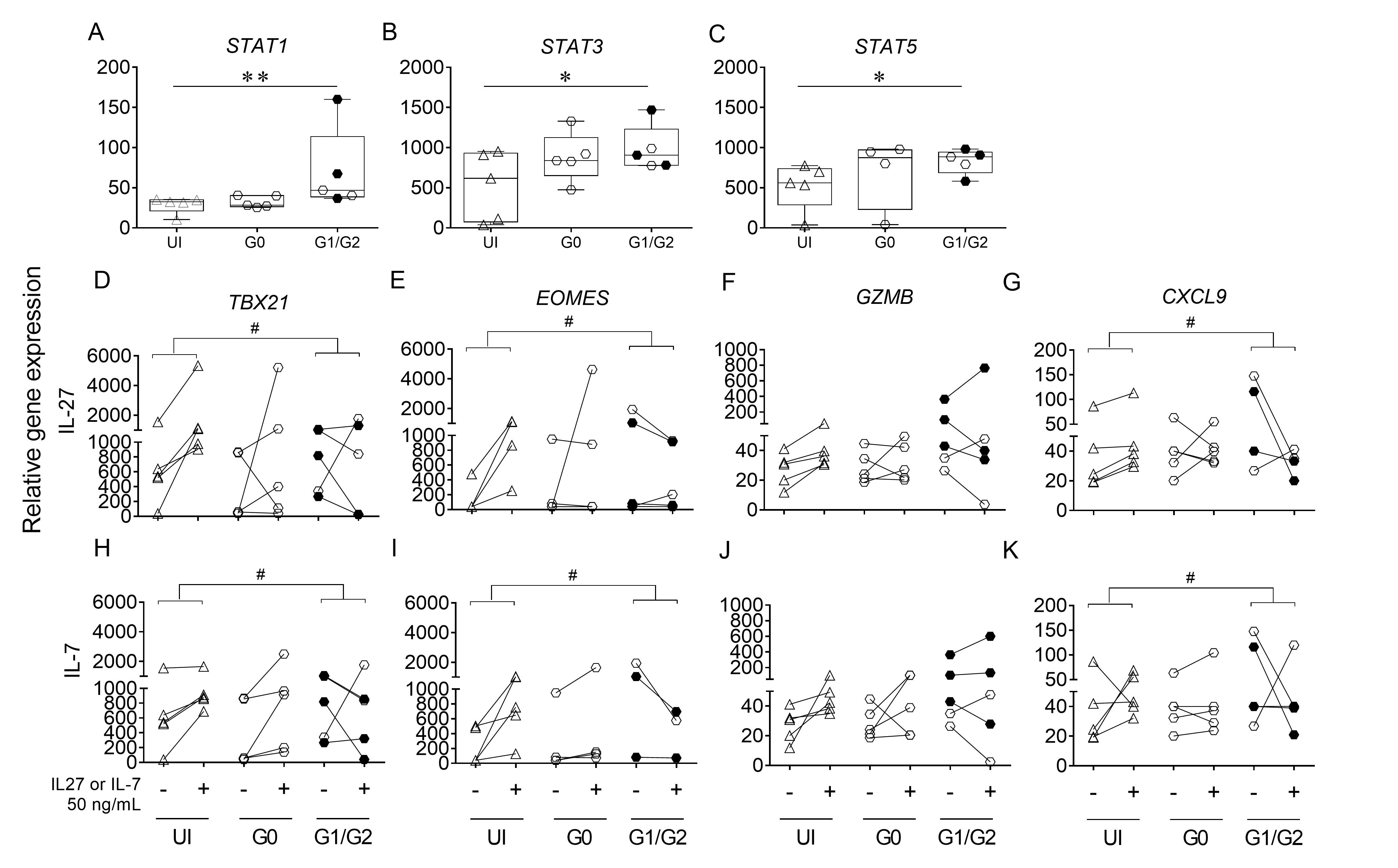

Supplement: S4 Fig — PBMCs were incubated for 24 h in AIM-V medium and subsequently subjected to 6 h of incubation in the presence or absence of 50 ng/mL IL-27 (D-G) or IL-7 (H-K). The total RNA was then isolated, cDNA was synthesized, and quantitative PCR was performed in all samples. Each symbol represents the constitutive gene expression of STAT1 (A), STAT3 (B) and STAT5 (C) or relative gene expression in unstimulated cells or after IL-27 or IL-7 in vitro stimulation of TBX21 (D, H), EOMES (E, I), GZMB (F, J), and CXCL9 (G, K), previously normalized to GADPH expression. Full black symbols represent data from subjects in the G1 clinical group. Medians are indicated by the horizontal lines; boxes indicate the 10–90 percentile range. * P < 0.05, ** P < 0.01 compared with uninfected subjects (UI) by Mann-Whitney test (A-C) # P < 0.05 show the difference between the ratio of cytokine-stimulated and unstimulated cultures by Mann-Whitney test. (TIF) [file pntd.0009473.s004.tif]

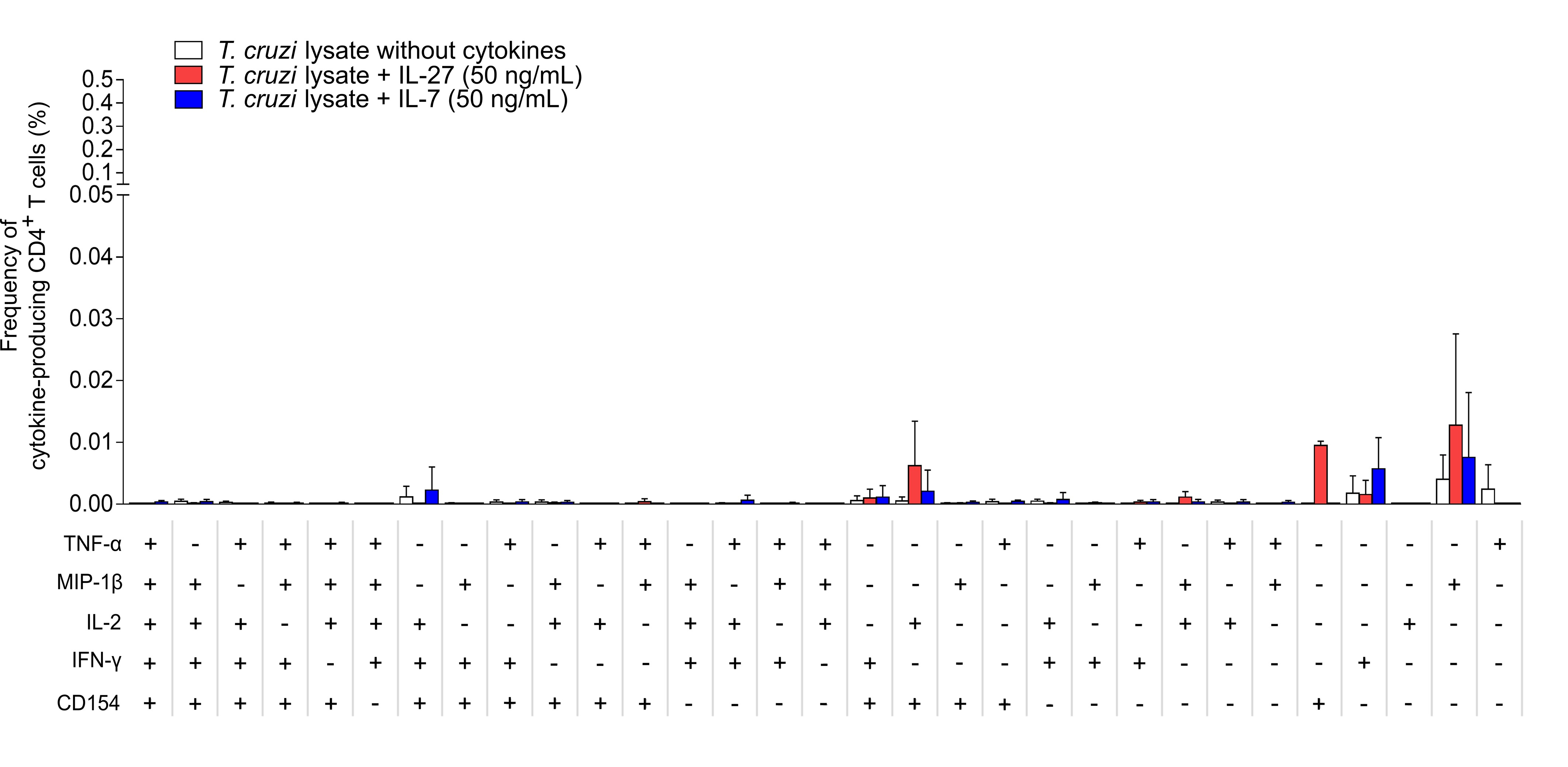

Supplement: S5 Fig — PBMCs of uninfected subjects (i.e., the UI group, n = 3) were stimulated with T. cruzi lysate preparation from the Brazil strain in the presence or absence of IL-27 (red bars) or IL-7 (blue bars) and analyzed using flow cytometry for the intracellular expression of TNF-α, MIP-1β, IL-2, IFN-γ and CD154 in CD4+ T cells. The cytokine coexpression profiles with one (1+), two (2+), three (3+), four (4+) and five (5+) functions were determined using the Boolean gating function of FlowJo software. Each bar represents the frequency of T. cruzi-specific (i.e., values obtained in cultures with only media were subtracted) CD3+CD4+ T-cell responses of each cytokine-producing population relative to the individual values of CD3+CD4+ for each subject. Data are shown as the mean and SD. (TIF) [file pntd.0009473.s005.tif]

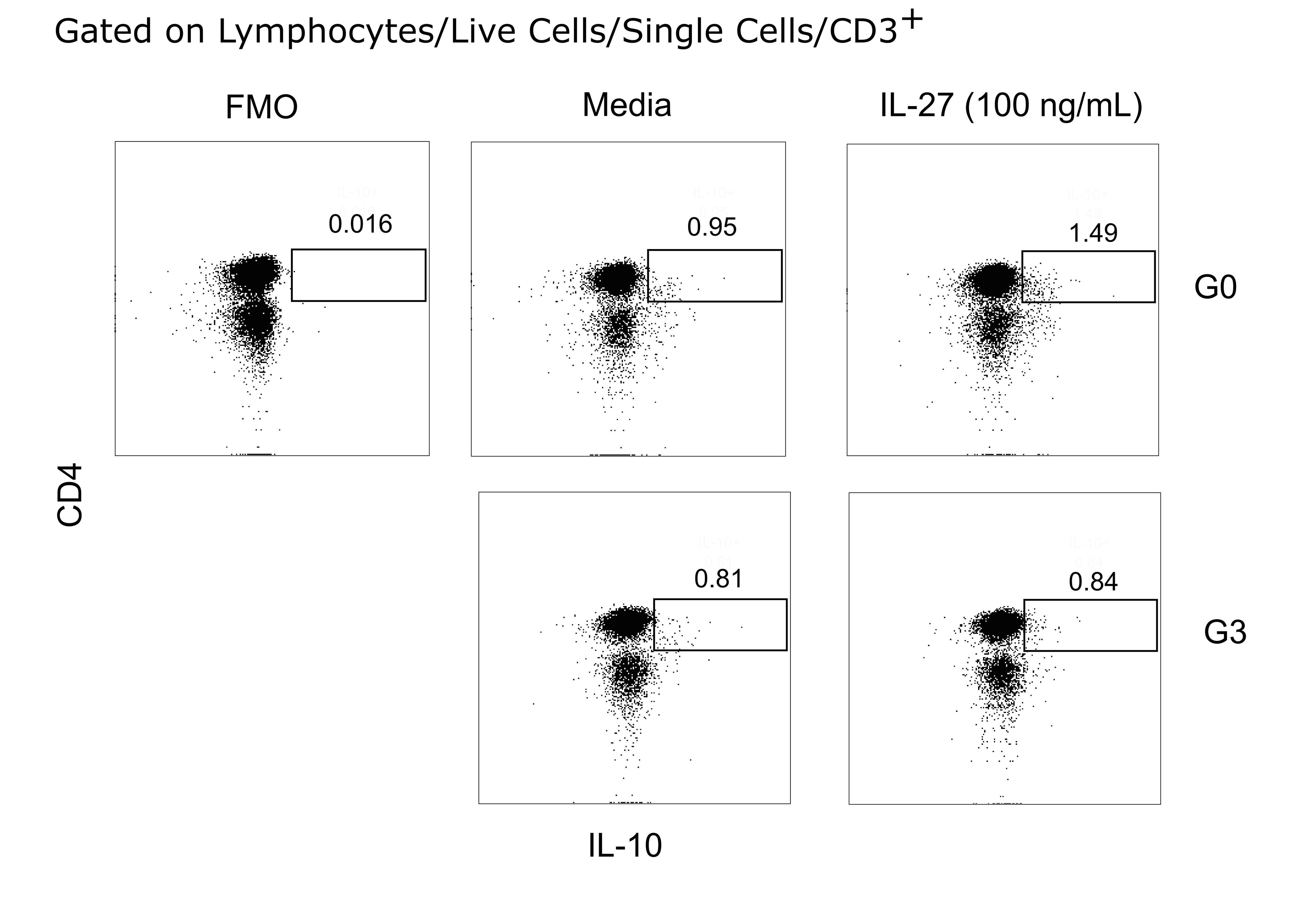

Supplement: S6 Fig — PBMCs collected from subjects with chronic Chagas disease with no signs of cardiac disease (i.e., the G0 group) or with myocardiopathy (i.e., the G3 group) were stimulated with 100 ng/mL of IL-27 or with media alone for 20 h, following which IL-10 production by CD4+ T cells was evaluated using flow cytometry. Representative dot plots for a G0 subject (upper panels) and a G3 subject (lower panels) are shown. IL-10+CD4+ T cells were gated according to FMO controls (upper left panel). (TIF) [file pntd.0009473.s006.tif]
